# A Classifier for Patient-Derived Colorectal Tumoroid Drug Sensitivity Using Confocal Imaging and Growth Rate Inhibition Metrics

**DOI:** 10.1158/2767-9764.CRC-25-0473

**Published:** 2026-03-04

**Authors:** Baard Cristoffer Sakshaug, Tonje H. Haukaas, Evelina Folkesson, Christa Ringers, Henri C.H. Bwanika, Ingrid A. Bergstrøm, Margrét S. Sigfúsdóttir, Hanne H. Trøen, Sigri B. Sperstad, Tore Stornes, Geir Klinkenberg, Torkild Visnes, Åsmund Flobak

**Affiliations:** 1 Department of Clinical and Molecular Medicine, Norwegian University of Science and Technology, Trondheim, Norway.; 2 Department of Biotechnology and Nanomedicine, SINTEF Industry, Trondheim, Norway.; 3 The Cancer Clinic, St Olav’s University Hospital, Trondheim, Norway.; 4 Department of Surgery, St. Olav’s University Hospital, Trondheim, Norway.

## Abstract

Patient-derived tumoroids have emerged as promising models for evaluating patient-specific responses to anticancer therapies, yet their clinical adoption remains limited. Although the reasons for this limited implementation are not fully elucidated, several are known and can be addressed: (i) lack of standardized protocols for tumoroid cultivation and drug exposure complicating cross-laboratory comparisons, (ii) labor- and time-intensive cultivation procedures conflicting with clinical guidelines for timely therapy initiation, (iii) prevalent use of destructive endpoint assays restricting subsequent analyses and proper growth rate correction, and (iv) poorly defined criteria for classifying tumoroid drug sensitivity that are not linked to clinical outcomes, leading to suboptimal treatment allocation in prospective studies. In this study, we developed two classifiers for assessing colorectal tumoroid sensitivity to oxaliplatin and SN-38 based on historic response rates for patients with colorectal cancer. Utilizing longitudinal, label-free confocal imaging, these classifiers offer a nondestructive method that corrects for growth rate variations and preserves patient-derived material for further analysis. Currently, these classifiers are undergoing evaluation in a prospective clinical trial to determine the feasibility of incorporating tumoroid-based drug screening into clinical decision-making. This approach lays the groundwork for next-generation molecular tumor boards, enabling anticancer treatment decisions informed by integrated functional assays and biomarkers.

**Significance:**

Patient-derived colorectal tumoroids can reveal which drugs are likely to induce a tumor response, but current protocols are slow and inconsistent. We developed rapid, nondestructive imaging-based classifiers for oxaliplatin and SN-38 that account for growth rate differences across patients, enabling reliable selection of oxaliplatin- versus irinotecan-based chemotherapy regimens in colorectal cancer.

## Introduction

Patient-derived tumoroids (PDT) are three-dimensional (3D) aggregates of malignant cells that are generated from surgical resections—or endoscopic or needle biopsies—of either primary or metastatic cancerous tumors. They are usually cultivated in specialized growth media and gel-like substances that allow them to grow in three-dimensions, which permits them to retain important characteristics of their parental tumor (1). Furthermore, several studies have demonstrated that PDTs can model—and in some cases predict—patient-specific responses to anticancer treatment (2–4). Despite their potential, only a few small-scale studies (5–8) and a single clinical trial (9) have used PDT drug screening assays to assign patients with colorectal cancer to anticancer treatment prospectively. In this clinical trial, 90 patients were included, and tumoroids were successfully generated for 44 patients, with at least one treatment suggested. Thirty-four patients were assigned treatment to the drug with the highest relative activity in their panel, 17 of whom met the primary endpoint of 2-month progression-free survival (PFS). No patient achieved a better response than stable disease (SD) at first RECIST evaluation.

There are several reasons for this slow transition from basic to clinical research (10, 11). Perhaps most important is the lack of standardized protocols for PDT establishment, maintenance, and drug–response assessment, which compromises the reproducibility and generalizability of the results as well as leading to variable culture success rates among laboratories. Although some studies have examined how protocol choices influence sample growth and culture success rate (12, 13)—including a previous study from our own laboratory (14)—most studies do not report on this (4).

The lengthy interval often required to generate PDT cultures and assess their drug sensitivity represents another challenge. Most groups do not report on the time-to-test, and those that do usually report a timeframe of several weeks (4)—too long to inform clinical decision-making (15). The delay largely stems from routine passaging of PDTs to expand their numbers before drug screening. To realize the potential of PDT technology for matching individual patients with effective anticancer therapy it is crucial to shorten the timeframe in which PDT drug screens are performed.

A further limitation is the widespread reliance on destructive endpoint ATP-based viability assays (4). Such readouts yield limited amounts of information—being single-parameter end point readouts—do not permit further analyses of samples post drug exposure, and cannot properly adjust for intersample growth rate variability, a factor known to skew drug-sensitivity estimates in *ex vivo* cellular cultures (2, 16). Implementing growth rate inhibition (GR) metrics (16)—or other methods that correct for disparities in proliferation-rate—can alleviate this artefact. Proper implementation of GR metrics requires repeated measurements of the same technical replicate, which is not possible using ATP-based viability assays.

Most clinical studies using PDTs to inform treatment decisions have done so using loosely defined drug-sensitivity thresholds that are not based on known clinical outcomes, for example allocating the drug with the “highest relative activity” among a panel of tested drugs (9), or similar formulations. A notable exception is the 2021 study by Ooft and colleagues (8), in which a PDT was classified as sensitive to a drug when it achieved a GR metric (16) of <0.1 at the highest tested drug concentration, although this classifier was not based on known clinical response rates either. Cross-drug comparison of PDT sensitivity using relative measures and cutoff values with unknown correlation to clinical outcomes is challenging because equipotency between the tested drugs is uncertain, as is the correlation between drug-specific tumoroid and patient sensitivity. Preferably, a drug-specific cutoff value that is correlated to known clinical outcomes should be selected for each drug to ensure reproducibility and clinical relevance, as well as reduce the impact of discretionary assessments.

The present study aimed to establish functional classifiers for drug sensitivity in PDTs that can be used to allocate patients with metastatic colorectal cancer to either FOLFOX [5-fluorouracil (5-FU), leucovorin, and oxaliplatin] or FOLFIRI (5-FU, leucovorin, and irinotecan) as first-line chemotherapy treatment while simultaneously addressing several of the aforementioned limitations in the field of tumoroid research. FOLFOX and FOLFIRI are the standard-of-care treatment for metastatic colorectal cancer, both achieving an objective response rate (ORR; ref. 17) of approximately 50%, and comparable overall survival (18). The triplet FOLFOXIRI—5-FU, leucovorin, oxaliplatin, and irinotecan—achieves a response rate of approximately 70% but is associated with worse toxicity (19). There is reason to believe that the improved ORR for FOLFOXIRI results not from drug synergies but simply from a higher probability of receiving at least one effective drug (20, 21). It is therefore reasonable to think that the response rates of both FOLFOX and FOLFIRI could be increased if one was able to predict patient sensitivity to the variable components in these regimens. No such method exists as of today, despite a substantial body of research into potential biomarkers (22).

To define sample drug sensitivity in a manner that is comparable across a wide range of drug susceptibility, one often uses dose–response modeling. This is a mathematical framework that uses regression analyses to estimate the mathematical relationship between a biological response and the concentration/level of an exposure or drug using only a selection of tested concentrations/levels and measured responses. Dose response modeling addresses the practical constraint of limited sample material, which again limits the number of concentrations/levels of exposure that can be tested. The fitted dose–response model will yield a dose–response curve (Supplementary Fig. S1) from which one can extract parameters of drug sensitivity that are commonly used in pharmacologic analyses, such as IC_50_ value (half-maximal inhibitory concentration) and area under the curve (AUC, an integrated measure of total sample sensitivity).

Here, we present the development of two classifiers for PDT sensitivity to the chemotherapeutic drugs oxaliplatin and SN-38 (the active metabolite of irinotecan)—the variable components of the chemotherapeutic regimens FOLFOX and FOLFIRI, respectively—that are based on known clinical response rates. We use continuous noninvasive image-based methods for evaluating PDT drug sensitivity—as opposed to destructive endpoint readouts—and combine image processing software, growth rate corrected metrics, and dose–response modeling to define the classifiers. Our approach allows for evaluation of PDT drug sensitivity within a week of sample acquisition, ensuring readouts within a clinically relevant timeframe.

## Materials and Methods

### Patient material

Tumor material from previously untreated patients undergoing surgical resection with curative intent of stages I to IV colorectal cancer at St. Olav’s University Hospital, Trondheim, Norway, was obtained following patients’ written informed consent. The study was approved by the Regional Committee for Medical and Health Research Ethics (ref. REK ID 10447). A total of 38 patients were sampled in the present study (Supplementary Table S1).

### Sample processing and cultivation

Samples were processed and cultivated as described previously (14), with minor modifications. Detailed cultivation procedures and a list of equipment and reagents can be found in Supplementary Materials and Supplementary Table S2. Briefly, following resection, the sample was collected in DMEM supplemented with 100 U/mL penicillin–streptomycin and ROCK inhibitor (10 μmol/L). The sample was minced into 1- to 2-mm pieces using scalpels and needles, followed by enzymatic digestion until the desired level of digestion is reached (usually 10–40 minutes) with collagenase type II (final concentration 1 mg/mL) in DMEM supplemented with ROCK inhibitor (10 μmol/L) at 37°C while being continuously stirred. Digestion was followed by serial filtration using 300- and 40-μm filters, from which the 40 to 300 μm fraction was kept for subsequent steps. After pelleting in a centrifuge (200–800 × *g*, 4°C, 5 minutes), the tumoroids were resuspended in a 1:1 mixture of a proteinaceous component (Matrigel) and a medium component [StemPro human embryonic stem cell serum-free medium (SFSCM) or DMEM/F-12 HEPES + 1% bovine serum albumin]. The tumoroid suspension was seeded in 50-μL droplets at a density of approximately 500 to 1,000 tumoroids/droplet in three 24-well plates (1 droplet per well, with tumoroids counted manually using a cell counting chip). Droplets were overlaid with 500 μL growth medium—either SFSCM or IntestiCult (commercially available organoid growth medium. See Supplementary Tables S3 and S4 for preparation of growth medium). Growth medium was replaced with drug-containing medium the following day. Samples were imaged approximately every other day for the duration of the experiment, which lasted from 7 to 14 days. For longer-lasting experiments, drug-containing medium was removed after 7 days and replaced with fresh growth medium, which was then changed every 3 to 4 days until the experiment ended. A sample was defined as successful when a mean relative total area (see below for definition) of at least 1.2 was reached in the untreated controls on day 7 of the cultivation period (23).

### Drug perturbation

See Supplementary Materials for details about drug exposure procedures. Briefly, the day after inclusion, growth medium was replaced with SFSCM or IntestiCult containing either oxaliplatin, SN-38 (the active metabolite of irinotecan), or the combination of the two in a fixed 5-step, 10-fold dilution series ranging from 0.012 to 120 μmol/L (for oxaliplatin) and 0.032 to 320 nmol/L (for SN-38). For the combination, we examined each concentration step in a fixed ratio, i.e., 0.012 μmol/L oxaliplatin and 0.032 nmol/L SN-38 in combination. Growth medium containing 0.5% DMSO was used as vehicle control. Each condition was examined in four technical replicates. Drug exposure lasted continuously for 6 to 7 days without medium change. For some samples drug-containing medium was then removed and replaced with fresh growth medium, and cultivation was continued for another 6 to 7 days.

### Imaging and image analysis

Imaging for monitoring sample growth was performed using an ImageXpress Micro Confocal High-Content Imaging System. Confocal imaging was performed at 37°C with a 2× objective. Each well was imaged in a Z-stack so that all tumoroids in the 3D gel droplets were captured in focus. Following imaging, each stack of images was converted into a 2D projection image using a “best focus” protocol. 2D projection images were exported as TIFF files to ImageJ (24), in which the image was binarized so that the tumoroid-covered area could be quantified. For details about the image analysis procedure, see Supplementary Materials.

### GR metrics

GR metrics were calculated using the method described by Hafner and colleagues (16):

$$GR\;metric\;\left(condition\right)\;=\;2^{\frac{\log2\left({Growth\;rate}_{condition}\right)}{\log2\left({Growth\;rate}_{control}\;\right)}}\;-\;1$$

We have defined growth rate as the relative total area on day 7 of the experiment which is calculated on a well-by-well basis by the following formula, in which x = 7:

$$Relative\;total\;area\;=\;\frac{{Total\;tumoroid\;covered\;area}_{day\;x}}{{Total\;tumoroid\;covered\;area}_{day\;1}}$$

See also Supplementary Fig. S2 for illustration. For every sample–drug pair, we first computed the GR value for each technical replicate by normalizing its growth rate to the mean of the untreated controls. After obtaining per-replicate GR values, we calculated their overall mean, avoiding bias that could arise from Jensen’s inequality (25).

For comparison, we also calculated the mean normalized relative total area of technical replicates per sample–drug pair, defined by the following formula:

$$Normalized\;relative\;total\;area\;=\;\frac{{Relative\;total\;area}_{drug\;concentration\;x}}{{Relative\;total\;area}_{DMSO-control}}$$

### Dose–response modeling and visualization

Hafner and colleagues (16) describes the following function for fitting GR data to a sigmoidal curve:

$$GR\left(c\right)\;=\;{GR}_{\inf}\;+\;\frac{1\;-\;{GR}_{\inf}}{1\;+\;{\left(c/{GEC}_{50}\right)}^{h_{GR}}}$$

In which *c* is the drug concentration, *GR*_*inf*_ is the lower asymptote, *GEC*_*50*_ is the concentration at which 50% of the observed effect is elicited, and *h*_*GR*_ affects the slope of the curve. This is mathematically equivalent to the three-parameter log-logistic function with the upper limit fixed at 1. We therefore used the DRC package (version 3.0-1; ref. 26) to fit the LL.3u function to our GR data on a sample-by-sample basis. We did the same for normalized relative total area.

The inferred dose–response curves were visualized by using fitted models to infer GR values or normalized relative total area per sample–drug combination from 1,000 uniformly distributed concentrations within the tested concentration range of each drug. We also calculated the mean and median inferred dose–response curves across all our samples, which are included in the visualizations.

The ED20/GR_50_ was calculated using the “absolute” method, which means that the value represents the concentration at which the inferred normalized relative total area or GR value is equal to 0.8 or 0.5, respectively. ED20 was chosen as parameter for the models fitted to normalized relative total area, as this was the highest level of inhibition that was reached by most of our samples. We also calculated area over the curve (AOC) for the inferred data generated above using an approximation of the trapezoidal rule:

$$AOC\;=\;\overset{n-1}{\underset{i=1}{\sum }}\left[\left(x_{i+1}\;-\;x_{i}\right)\;\times \;\frac{\left(1\;-\;y_{i}\right)\;+\;\left(1\;-\;y_{i+1}\right)\;}{2}\right]$$

In which x represents the uniformly distributed concentrations and y represents the corresponding inferred biological response. AOC was used instead of AUC, as normalized GR curves can have negative values, making AOC more intuitive to interpret. Dose–response modeling and visualization were performed using R (version 4.4.2) and the DRC package (version 3.0-1; ref. 26).

### Statistical analysis

Normality of continuous variables was evaluated using the Shapiro–Wilk test and by generating Q–Q plots. For continuous variables, the Wilcoxon rank-sum test was used for non-normally distributed values, whereas the two-sample *t* test was used for normally distributed values. Fisher exact test was used for categorical variables. McNemar test was used in the case of correlated proportions. Outcomes were reported as *P* values with confidence intervals (CI) representing either the absolute difference between the groups (continuous variables) or odds ratio (OR; categorical variables). CIs or interquartile ranges (IQR) are reported where applicable. Bootstrapping was performed to estimate the precision of point estimates. All analyses were performed using R version 4.4.2. A complete list of packages used can be found in Supplementary Table S5, and the code used is supplied in the supplementary materials (Supplementary R Script).

### Simulated samples

Ten samples were defined to have negative control relative total area (see above for definition) of 10, 5, 4, 3, 2, 1.9, 1.8, 1.7, 1.6, and 1.5 on day 7 and were named thereafter. The simulated samples were “exposed” to the same concentrations of oxaliplatin and SN-38 as our live samples. We defined that each increase in concentration should confer a 20% reduction in day 7 relative total area compared with vehicle control, resulting in the highest concentration achieving a 100% reduction compared with vehicle control, i.e., complete cytostasis. Each simulated sample had the same number of technical replicates as the live samples, although variability was not introduced in the technical replicates. We only generated data for days 1 and 7 for the simulated samples.

We calculated GR metrics for each simulated sample (see above for definition) and used both the GR metrics and normalized relative total area to fit the log-logistic three-parameter model. We generated dose–response curves as previously described and extracted GR50/ED20 for each simulated sample and compared this with the median GR50/ED20 from our library of live samples.

## Results

### Overview of the study

**Figure 1.**
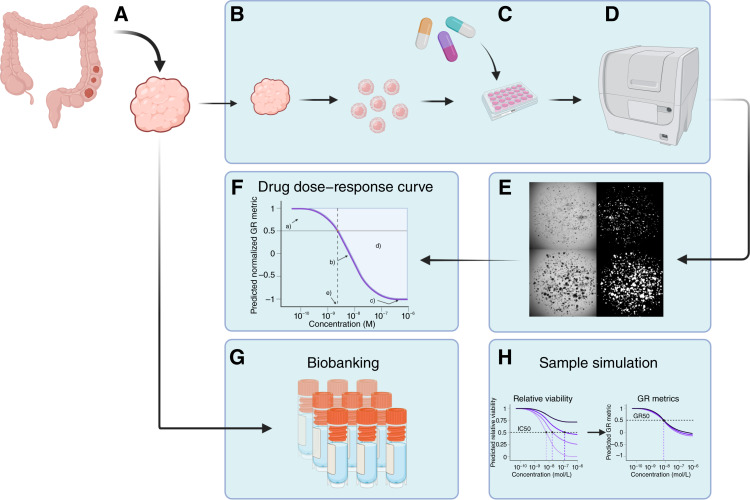
Overview of the study. **A,** Collection of colorectal cancer sample material; surgical biopsies were used in the present study. **B,** Processing of sample and seeding as tumoroids. **C,** Addition of drugs. **D,** Confocal imaging of samples, repeated several times through the course of the experiment. **E,** Binarization of confocal imaging in order to quantify tumoroid-covered area. **F,** Generation of dose–response curves from parameters derived from binarized confocal images. **G,** Biobanking of leftover sample material. **H,** Simulation of tumoroid samples to investigate how growth rate affects evaluation of sample sensitivity using relative viability and GR metrics. Dose–response curves are not from actual simulations. [Created in BioRender. Sakshaug, C. (2026) https://BioRender.com/qg9jkww.]

A graphical overview of the present study can be seen in Fig. 1. A total of 38 patient samples were included, 16 of which were categorized as successful. Nineteen samples failed to reach a mean relative total area of 1.2 in control wells by experimental day 7 and were excluded from analysis, 16 of which did not grow at all, and three of which grew too little. Three samples were unfortunately subject to experimental errors, although they displayed sufficient growth in at least one condition. Excluding the three erroneous samples, this gives a culture success rate of 16/35 (45.7%; Supplementary Table S1). The patients representing the two groups (successful vs. unsuccessful samples) were compared across several descriptive statistics (Supplementary Table S6). The successful samples were derived from patients with higher clinical stage (*P* value = 0.046) and who more often received adjuvant therapy (*P* value = 0.032, 95% CI for OR = 1.01–522). Left-sided colon cancer resulted in more successful samples as compared with right-sided colon cancer and rectal cancer (*P* value = 0.026), and there was a slight trend toward successful samples more frequently being derived from male patients as compared with females (*P* value = 0.15, 95% CI for OR = 0.69–27.3).

### Dose–response relationship manifests shortly after drug exposure

We aimed to determine whether PDTs could be cultured, exposed to drugs, and classified for sensitivity within a clinically actionable window—without passaging the sample. Passaging prolongs culture time and could promote clonal selection that leads to divergence from parental tumor biology (27) and conflicts with stringent clinical guidelines for treatment initiation (15).

To define the shortest workable window, we tracked the temporal evolution of the dose–response curves of seven PDT samples (samples 1, 3, 7, 8, 10, 12, and 13). Each sample was cultured for 13 to 14 days: 1 day without drug, 6 to 7 days with drug, and finally 6 to 7 days after drug treatment. Confocal imaging was performed repeatedly. GR metrics were calculated for each imaged day, and dose–response curves were generated where possible using the LL3.u model.

**Figure 2.**
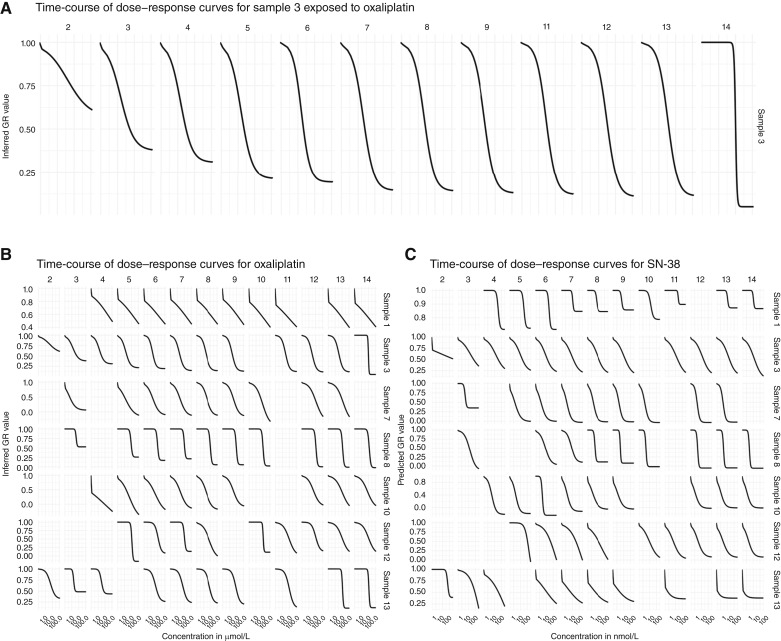
Time-course analysis of sample library dose–response. **A,** Time-course of dose–response curves for sample 3 exposed to oxaliplatin. **B** and **C,** Time-course of dose–response curves for seven samples cultivated for 13–14 days. Blank spaces indicate either missing data from that day or that a curve could not be fit to data from that day.

Figure 2 and Supplementary Fig. S3 illustrates that dose-dependent drug responses could be detected as early as 1 day after drug exposure and that all samples displayed dose–response relationships that could be modeled by day 5 of the cultivation period, with varying goodness-of-fit. Curve shapes remained largely stable from this timepoint, with some variation in maximal response and curve steepness. To balance assay speed and allowing the tumoroids enough time in culture to reach a sufficient relative total area in vehicle controls for dose–response modeling (see “Materials and Methods”), we selected day 7 of the cultivation period (6 days after drug exposure) as the timepoint for constructing our drug-sensitivity classifiers.

### Sample library demonstrates clinically relevant GR50 values

ORRs for first-line chemotherapy in metastatic colorectal cancer show that roughly 50% of patients will respond to either FOLFOX or FOLFIRI—which are considered equivalent options (15, 18)—and there is reason to believe that the increased ORR of FOLFOXIRI is due to the patient being more likely to receive one or more drug that is effective against their tumor (20, 21). As FOLFOX and FOLFIRI differ only in their backbone drug—oxaliplatin versus irinotecan—stratifying patients and allocating treatment according to sensitivity to these variable agents could potentially increase the ORR to levels similar to FOLFOXIRI.

**Figure 3.**
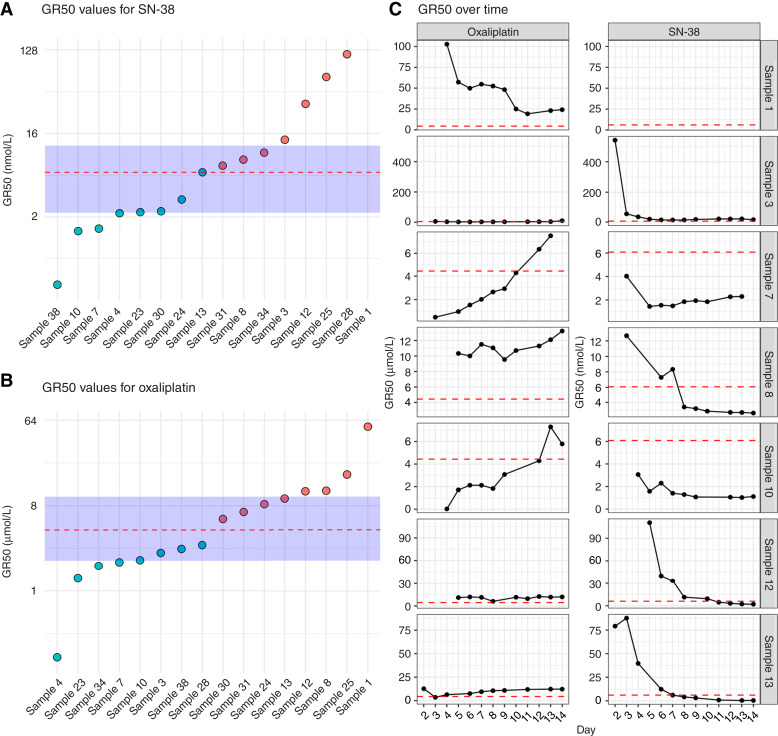
**A** and **B,** Point estimate of sample library GR50 on day 7; the *y*-axis is log-transformed. The dotted red line represents the median GR50, and the blue area represents the IQR. Dot color indicates sample sensitivity according to the median: red = resistant and blue = sensitive. **C,** Time-course analysis of point estimate GR50 for seven samples cultivated for a total of 13–14 days. The dotted red line represents the median GR50 of all 16 samples on day 7. Missing data points indicate either missing data from that day or that a dose–response curve from that specific day could not be fitted. *Y*-axes are linear.

We therefore exposed our tumoroid samples to single-agent treatment with oxaliplatin and irinotecan and fit dose–response curves for each sample–drug combination (see “Materials and Methods”). This resulted in 32 unique dose–response curves, with varying goodness-of-fit (Supplementary Table S7). We then extracted GR50—the dose require to reduce normalized GR metric to 0.5—for single-agent treatment with oxaliplatin and SN-38 for all sample–drug combinations. Only one sample–drug combination did not reach this level of inhibition (sample 1 + SN-38). The median GR50 for oxaliplatin was 4.44 μmol/L [Fig. 3A; 95% CI, 2.12–9.50 μmol/L; IQR = 2.10–9.96 μmol/L, unscaled median absolute deviation (MAD) = 2.28 μmol/L], close to the reported *in vivo* peak plasma concentration (*C*_max_ = 4.96 μmol/L; ref. 28). For SN-38, the median GR50 was 6.08 nmol/L (Fig. 3B; 95% CI, 2.23–11.8 nmol/L; IQR = 2.23–11.80 nmol/L, unscaled MAD = 3.89 nmol/L), substantially lower than the reported *in vivo C*_max_ (143 nmol/L; ref. 28).

We then tracked GR50 longitudinally in seven extended cultures (samples 1, 3, 7, 8, 10, 12, and 13) that were grown for 13 to 14 days. For some samples, GR50 for oxaliplatin rose moderately, whereas GR50 for SN-38 tended to decline (Fig. 3C). For most samples, however, GR50 stabilized around day 7 to 8 of the cultivation period, although several of these estimates are burdened with considerable uncertainty (Supplementary Fig. S4). When applying day 7 median GR50 values for all 16 evaluable samples, samples 1, 3, 7, 8, 10, 12, and 13 were consistently classified as either sensitive or resistant in eight of 14 sample–drug combinations from day 7 and onward (Supplementary Fig. S5).

### Using GR metrics reduces the impact of growth rate on evaluation of sample sensitivity

Sample growth rate and doubling time are known to bias drug-sensitivity estimates, in which faster-growing samples tend to be systematically evaluated as more sensitive than slower-growing samples (2, 16). In our PDT library, relative total area in control wells on day 7 of the cultivation period ranged from 1.07 to 4.43, indicating substantial heterogeneity in growth rate (Supplementary Fig. S6). To examine whether any growth rate bias was present in our data, we divided our 16 PDT samples into “fast” and “slow” halves based on mean relative total area in untreated controls on day 7 and fitted dose–response curves to normalized relative total area and GR metrics separately.

**Figure 4.**
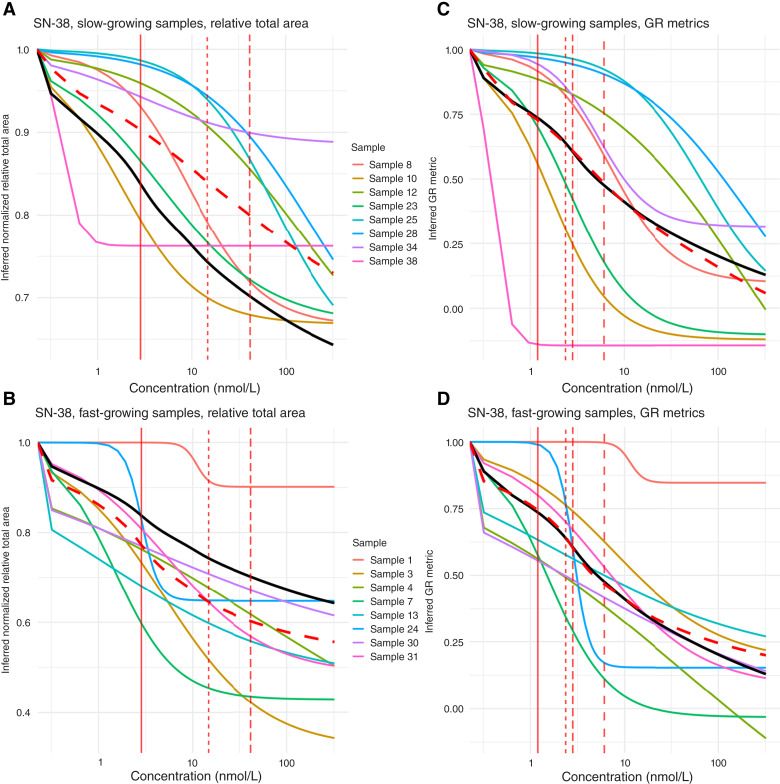
Dose–response curves for SN-38 generated using the log-logistic 3-parametric model fitted to either relative total area (**A** and **B**) or GR metrics (**C** and **D**). The *x*-axis displays drug concentration in nanomolar (nmol/L), and the *y*-axis displays inferred response (normalized relative total area/GR metric). Solid black line indicates the library mean inferred response across both fast- and slow-growing samples per readout, and dashed red line indicates the mean inferred response for only the fast- or slow-growing samples, respectively, per readout. Vertical red lines indicate median ED values for increasing levels of inhibition (20%, 30%, 40%, and 50%) across both fast- and slow-growing samples. **A,** Dose–response curves of the eight slowest-growing samples fitted to relative total area. **B,** Dose–response curves of the eight fastest-growing samples fitted to relative total area. **C,** Dose–response curves of the eight slowest-growing samples fitted to GR metrics. **D,** Dose–response curves of the eight fastest-growing samples fitted to GR metrics.

When models were fitted to normalized relative total area, the eight slowest samples produced curves that clustered above the library mean, whereas the eight fastest clustered below (Fig. 4A and B; Supplementary Fig. S7A and S7B), implying that slow-growing samples would be considered less sensitive than fast-growing. Conversely, models fitted to GR metrics displayed a more balanced distribution around the library mean across growth rates (Fig. 4C and D; Supplementary Fig. S7C and S7D). Consistent with these results, only 3/8 and 2/8 slow-growing cultures were classified as sensitive to oxaliplatin and SN-38, respectively, using library median ED20 as threshold, whereas when using median library GR50 as threshold, 5/8 and 3/8 of the slowest-growing samples were classified as sensitive to oxaliplatin and SN-38, respectively (Supplementary Figs. S8 and S9). When using McNemar test for correlated proportions, there were no significant differences between how median ED20 and GR50 classified the slow- and fast-growing samples’ sensitivity to oxaliplatin and SN-38.

Therefore, to further strengthen our decision-making, we simulated data from 10 idealized samples with identical relative drug sensitivity but varying growth rates. Live sample median GR50 was more consistent in classifying simulated sample sensitivity than median ED20 (Supplementary Figs. S10 and S11). We also saw that estimated simulated sample GR50 seemed to stabilize with decreasing growth rate, whereas estimated ED20 increased exponentially (Supplementary Figs. S10 and S11). Most of our live samples exhibit a mean relative total area ≤2 in untreated controls on day 7 (Supplementary Fig. S6), which suggests that the use of GR metrics will minimize any growth rate–induced drug-sensitivity bias in our samples and provide a more reliable foundation for generating our drug-sensitivity classifiers than normalized relative total area.

### Integrated sensitivity measure gives complementary insights into sample sensitivity

Although GR50 is intuitive and easily comparable across samples—and could, in theory, serve as a two-point classifier that inspects only vehicle control and median GR50—it is sensitive to curve shape and experimental noise, being less reliable for shallow and nonsigmoidal curve shapes (29), and cannot be computed for samples that do not reach a normalized GR level of 0.5.

Because many samples in our library produce dose–response curves with gradual, rather than steep, slopes (Fig. 4; Supplementary Fig. S7), we included AOC (16) into our evaluation of sample sensitivity (see “Materials and Methods”; Supplementary Fig. S1). In contrast to point estimates such as GR50, AOC integrates inferred growth inhibition across the entire concentration range, can be calculated for every sample regardless of the maximum achieved effect, and is less sensitive to curve shape and experimental noise (30, 31). AOC is the mathematical complement to AUC, and therefore an equivalent measure. A higher AOC indicates a more sensitive sample.

**Figure 5.**
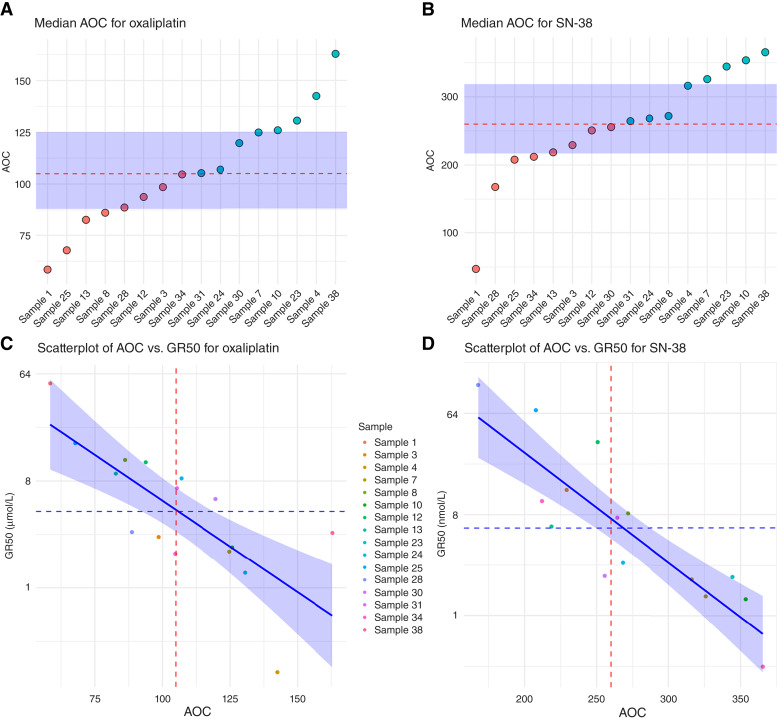
**A** and **B,** Point estimates of sample library AOC. The dotted red line represents median AOC, and the blue area represents the IQR. Dot color indicates sample sensitivity according to the median, with red indicating resistant and blue indicating sensitive. **C** and **D,** Correlation plots of GR50 and AOC. The *y*-axis is log-transformed, and the *x*-axis is linear. The dotted blue line indicates the median GR50, the dotted red line indicates the median AOC, and the whole blue line indicates linear regression of log_2_(GR50) and AOC with CIs. GR50 is in μmol/L for oxaliplatin (left) and nmol/L for SN-38 (right).

Across our 16 successful samples, the median AOC for oxaliplatin was 105 (95% CI, 88.7–124.8; IQR = 88–125) and for SN-38 was 260 (95% CI, 218.6–316.2; IQR = 220–320; Fig. 5A and B). Median GR50 and AOC agreed on sample sensitivity in 10/16 cultures for oxaliplatin and 13/16 cultures for SN-38 (Fig. 5C and D; Supplementary Fig. S12). Regression analysis of log_2_(GR50) against AOC revealed a strong correlation, with correlation coefficients of −0.76 for oxaliplatin and −0.87 for SN-38.

## Discussion

PDTs have been suggested as patient-specific cancer models that can replicate *in vivo* tumor biology and retrospectively mirror responses to anticancer treatment (1–4)—or even prospectively predict them (5–9). They could therefore become central in next-generation tumor boards, in which anticancer treatment is allocated based on patient-specific biomarkers and functional assays, instead of group-level data and empirical decision-making. However, most prospective PDT-guided studies remain small and report mixed predictive accuracy (5–9). Although the issue is multifaceted, major actionable concerns include (i) the lack of standardized and optimized protocols for PDT culture, drug exposure, and sensitivity readouts; (ii) time requirements outside of clinical decision-making windows; (iii) the use of destructive endpoint readouts that do not allow correction for growth rate variability or further examination of surviving PDTs; and (iv) the lack of drug-specific sensitivity cutoffs with known correlation to clinical outcomes.

Image-based and growth rate corrected readouts are uncommon in colorectal cancer PDT research (2, 7–9, 32–34) and have not yet been utilized in combination. They have, however, been jointly applied to PDTs derived from pancreatic ductal adenocarcinoma with normalized organoid growth rate (NOGR) metrics, as recently reported by Deben and colleagues (23). The NOGR metric expands the 2D methods developed by Hafner and colleagues (GR metrics; ref. 16) and Gupta and colleagues [normalized drug response (NDR) metric; ref. 35] to three-dimensions, and the NOGR metric is highlighted as more accurate than both GR metrics and NDR metrics in classifying cytotoxic responses in slow-growing tumoroids and cytostatic responses in fast-growing tumoroids. We have used GR metrics as calculation of both NDR and NOGR metrics require positive control wells—something our setup does not include.

Most protocols for establishing and cultivating colorectal cancer PDTs involve single-cell seeding and passaging steps before drug screening is performed. We instead seed our biopsies as clusters of tumor cells, so drug exposure can begin almost immediately. Our time-course analysis indicates that robust dose–responses can be observed as early as the day after drug exposure and is present in all samples by day 6 to 7 of the cultivation period (Fig. 2). GR50 values seem to stabilize around the same time (Fig. 3C; Supplementary Figs. S3 and S4), indicating that drug sensitivity can be reported as early as 1 week after biopsy acquisition—well inside clinical decision windows (15), while simultaneously allowing the majority of the samples to reach a sufficient relative total area in vehicle controls to perform dose–response modeling. We therefore selected day 7 for generating our drug-sensitivity classifiers. Additionally, cluster seeding limits the possibility of clonal drift linked to single-cell expansion and prolonged cultivation periods (27), although the presence of such drift is not certain (36).

Most PDT studies still rely on destructive endpoint readouts that prevent proper growth rate normalization (4). Our findings indicate that omitting growth rate correction systematically underestimates the drug sensitivity of slow-growing tumoroids (Fig. 4; Supplementary Figs. S8–S11)—a critical flaw, given that most PDTs proliferate slowly within clinically relevant timeframes (Supplementary Fig. S6). By using label-free imaging techniques, tumoroid growth can be evaluated longitudinally, facilitating proper growth rate correction on a well-by-well basis. The resulting images allow calculation of GR metrics and interpolation of GR50 and AOC, both of which could serve as classifiers for drug sensitivity in PDT cultures (Fig. 5). Our analysis does, however, assume that the tumoroid-covered area accurately reflects the number and viability of the cultured cells, which is not necessarily true. Both oxaliplatin and SN-38 might induce morphologic and metabolic alterations in the tumoroids, which could decouple the assumed correlation between tumoroid area and cell number and viability. However, this correlation is not strictly necessary for correlating tumoroid-covered areas to patient outcomes.

Prospective studies using colorectal cancer PDTs to inform treatment decisions have done so using flexible and poorly defined cutoffs that are not correlated to known clinical outcomes—such as allocating the drug with the highest relative activity (9) or defining a certain GR value as cutoff for sensitivity without knowing the drug-specific correlation between tumoroid and corresponding patient drug sensitivity (8). We have developed drug-specific classifiers for sensitivity to oxaliplatin and SN-38 (Figs. 3 and 5) that are based on known clinical outcomes for the chemotherapeutic regimens FOLFOX and FOLFIRI, of which oxaliplatin and SN-38 are the variable components. The historic ORR for both FOLFOX and FOLFIRI is approximately 50% (18), and using the library median GR50 and AOC as sensitivity cutoffs for oxaliplatin and SN-38 could therefore allow identification of the patients most sensitive to each variable component as proxies for sensitivity to the combination regimens. We hypothesize that allocating FOLFOX or FOLFIRI based on our PDT-based classifiers could raise the probability of assigning effective therapy to levels similar to FOLFOXIRI—around 70%. Although we could not retrospectively compare the responses of tumoroids and corresponding patients in this study—as none of the patients received the appropriate treatment—they will be validated and further refined through ongoing research in our group.

The historic ORR for the triplet FOLFOXIRI suggests that approximately 70% of patients should be sensitive to either FOLFOX or FOLFIRI, a proportion of which will be sensitive to both and another proportion to only one of the two. Applying the GR50 cutoff, 7 of 16 PDT samples displayed discordant sensitivities to oxaliplatin and SN-38, whereas only 2 of 16 differed when using the AOC cutoff (Supplementary Fig. S12). Thus, several samples in our library were classified as either sensitive or resistant to both drugs, regardless of which cutoff was used. Additionally, the two classifiers yielded discordant sensitivities for a subset of drug–sample combinations (Supplementary Fig. S12), which could complicate treatment allocation. We therefore aim to rely on AOC as the main classifier, as there are indications that AOC is better correlated with patient responses than GR50 (2), and use GR50 as a supporting readout. Should a sample be classified as sensitive or resistant to both drugs, we will allocate the treatment with the largest or smallest relative distance from the median, respectively. Ultimately, a prospective clinical trial will be required to determine whether our classifiers can distinguish responders from nonresponders and guide treatment choices when both drugs—or neither—seem effective.

Although our method advances PDT-guided therapy approaches toward routine clinical practice, several limitations remain. First, our culture success rate of 46% is low compared with other laboratories (4). There are indications that culture success correlates with patient clinical stage, as well as tumor sidedness (Supplementary Table S6), but out cohort is small, and the findings are therefore uncertain. It is plausible that the aggressiveness of a tumor—indicated by higher clinical stage—and disparities between right- and left-sided tumors (37) affect PDT culture success rate, which warrants further investigation. However, we believe suboptimal tissue handling, protocol variables, and growth medium composition to be the more likely culprits. This has been emphasized by other researchers as well (38). Second, in the present study, we have been working with surgical biopsies and therefore abundant sample material. Patients with metastatic disease will not always undergo surgical removal of the primary tumor, and core-needle biopsies will often be a more practical alternative. Needle biopsies can yield smaller amounts of sample material than surgical biopsies, and our protocols must be miniaturized to accommodate this. Finally, neither the AOC nor the GR50 classifier has been prospectively validated in a clinical cohort. We have therefore launched a clinical trial in which we will allocate patients with metastatic colorectal cancer to first-line treatment with either FOLFOX or FOLFIRI based on tumoroid sensitivity to oxaliplatin and SN-38. The primary endpoint is to evaluate the feasibility of using the methodology in a clinical setting, in which we attempt to deliver drug sensitivity reports within a clinically relevant timeframe. Secondary endpoints include evaluating how using PDTs to guide therapy choices affects treatment efficacy in terms of ORR and PFS (CTIS number: 2024-517677-25-00).

### Conclusions

In the present study, we have cultured PDTs from 16 patients with colorectal cancer, exposed them to oxaliplatin and SN-38, and evaluated their drug sensitivity using noninvasive, longitudinal, label-free imaging within 1 week of sample acquisition. We adapted GR metrics as defined by Hafner and colleagues (16) to our image-based readouts, built dose–response models, and defined two drug-sensitivity classifiers—median GR50 and AOC—that are less affected by growth rate variability than conventional readouts. These classifiers could serve as functional surrogates for patient sensitivity to oxaliplatin and SN-38—the variable components of FOLFOX and FOLFIRI—and represent an important step toward future molecular tumor boards, in which PDT assays and other biomarkers can be combined with clinical expertise to further individualize cancer therapy.

## Supplementary Material

Supplementary Table S1 Patient characteristics of individual samples.

Supplementary R Script R script for data analysis and generation of figures.

Supplementary Figure S1 Illustration of sigmoidal dose-response curve

Supplementary Figure S2 Illustration of relative total area

Supplementary Figure S3 Time-course analysis of sample library dose-response with raw measurements and standard deviation included.

Supplementary Figure S4 Time course analysis of point estimate GR50 for 7 samples cultivated for a total of 13-14 days.

Supplementary Figure S5 Classification of Samples 1, 3, 7, 8, 10, 12, and 13 as either sensitive or resistant to oxaliplatin and SN-38 for the total duration of the experiment, according to the median GR50 of all 16 samples on day 7 of the experiment.

Supplementary Figure S6 Growth rate (relative total area) of negative controls in all growing samples.

Supplementary Figure S7 Dose response curves for Oxaliplatin generated using the log-logistic 3-parametric model fitted to either relative total area or GR-metrics.

Supplementary Figure S8 Classification of the eight slowest growing samples as either sensitive or resistant to oxaliplatin and SN-38 at day 7 of the experiment according to either median ED20 or median GR50.

Supplementary Figure S9 Classification of the eight fastest growing samples as either sensitive or resistant to oxaliplatin and SN-38 at day 7 of the experiment according to either median ED20 or median GR50.

Supplementary Figure S10 Barplot of estimated GR50 of simulated samples.

Supplementary Figure S11 Barplot of estimated ED20 of simulated samples.

Supplementary Figure S12 Sensitivity of all samples as sensitive or resistant to oxaliplatin and SN-38 using median GR50 and median AOC.

Supplementary Material Detailed description of methods.

Supplementary Table S2 List over materials and reagents used.

Supplementary Table S3 Reagents used for preparation of SFSCM.

Supplementary Table S4 Reagents used for preparation of IntestiCult.

Supplementary Table S5 List of R-packages used.

Supplementary Table S6 Descriptive statistics of patient material.

Supplementary Table S7 Residual standard error (RSE) of all sample-drug combinations.

## Data Availability

All data generated and analyzed in the present article can be made available at reasonable request to the corresponding author.
