# Supplementary material for: A Classifier for Patient-Derived Colorectal Tumoroid Drug Sensitivity Using Confocal Imaging and Growth Rate Inhibition Metrics
Source: Cancer Res Commun. 2026 Mar 4;6(3):466–76. doi: 10.1158/2767-9764.CRC-25-0473 (PMC13012007; doi:10.1158/2767-9764.CRC-25-0473)
Supplement: Supplementary Figure S1 — Illustration of sigmoidal dose-response curve [file crc-25-0473_supplementary_figure_s1_suppsf1.docx]

**
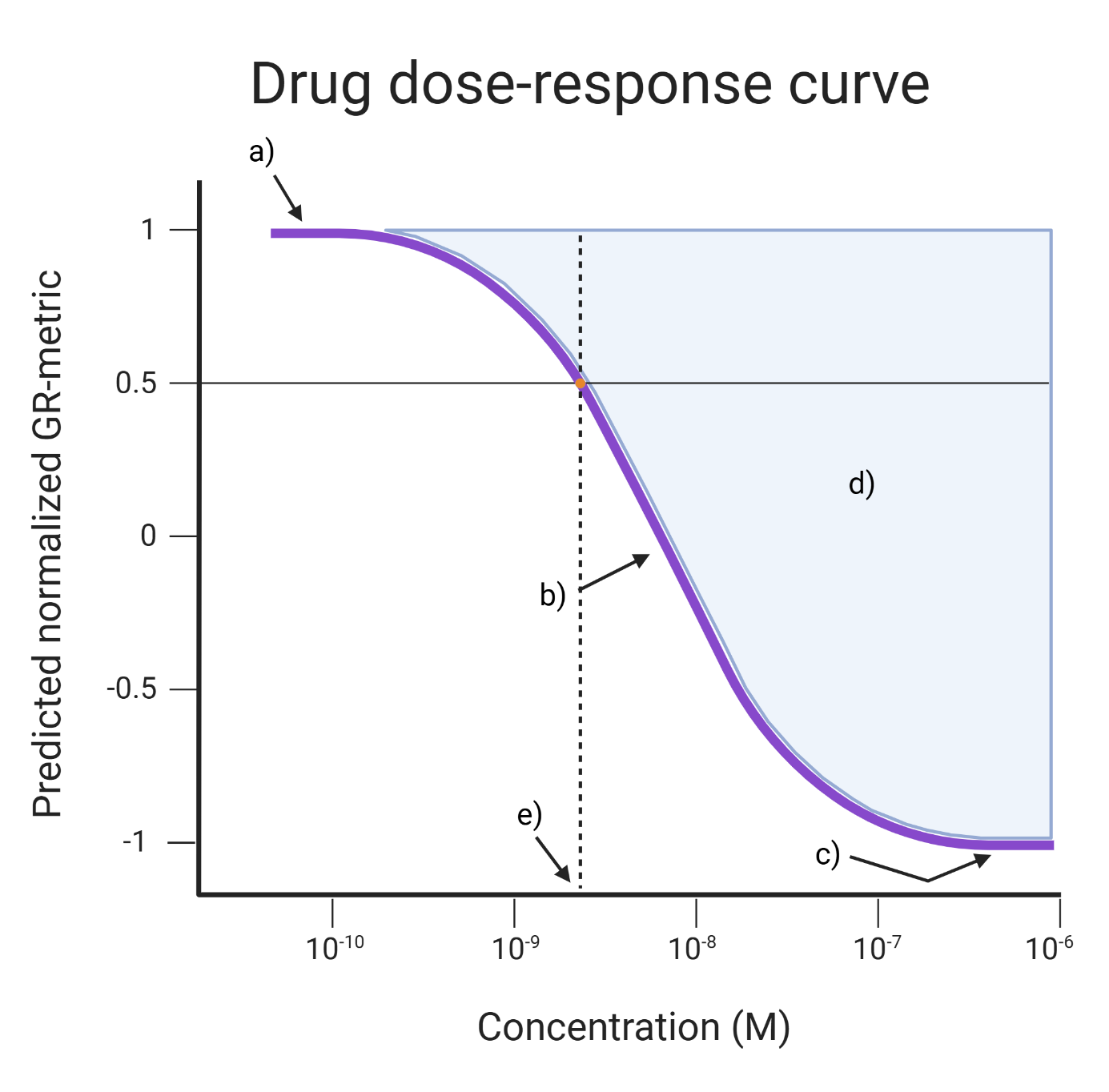
**

**Supplementary Figure S1.** **Illustration of sigmoidal dose-response curve.** **a)** Upper asymptote, usually defined as the normalized GR-metric of the negative control, which in our material corresponds to the untreated tumoroids. **b)** Inflection point, i.e. the steepest point of the curve, and the point where 50% of the observed drug effect is elicited. **c)** Lower asymptote, i.e. the maximum observed drug effect. **d)** Area over the curve (AOC), an integrated measure of sample sensitivity. **e)** GR50, the concentration at which inferred normalized GR-metric is equal to 0.5 of untreated tumoroids, a point estimate of sample drug sensitivity. Created using BioRender (2025).
