# Supplementary material for: A Classifier for Patient-Derived Colorectal Tumoroid Drug Sensitivity Using Confocal Imaging and Growth Rate Inhibition Metrics
Source: Cancer Res Commun. 2026 Mar 4;6(3):466–76. doi: 10.1158/2767-9764.CRC-25-0473 (PMC13012007; doi:10.1158/2767-9764.CRC-25-0473)

**Supplementary Figure S2.** Illustration of relative total area. The top two images are from day 1 of the experiment, and the bottom two images are from day 7 of the experiment. The left-most images are raw images, while the right-most are binarized images. The white portion of the images on the right side represents the tumoroid-covered area that is quantified using ImageJ and subsequently used to calculate growth rate/relative total area.


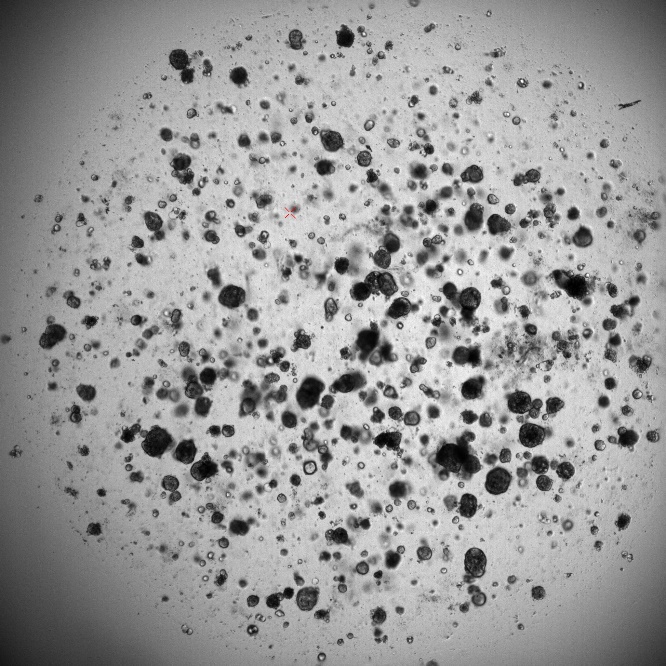

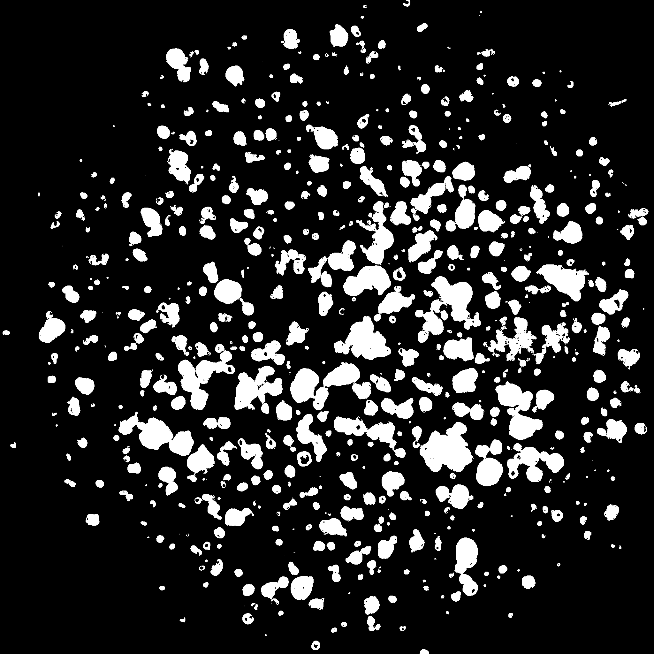

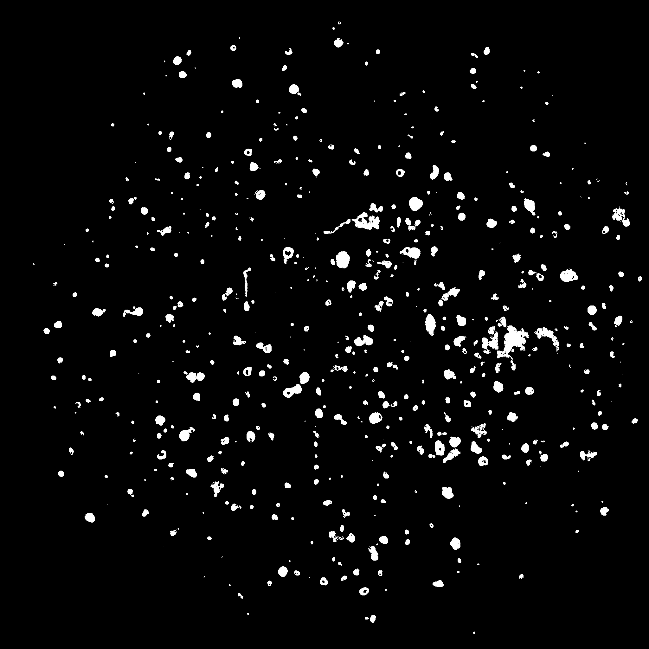

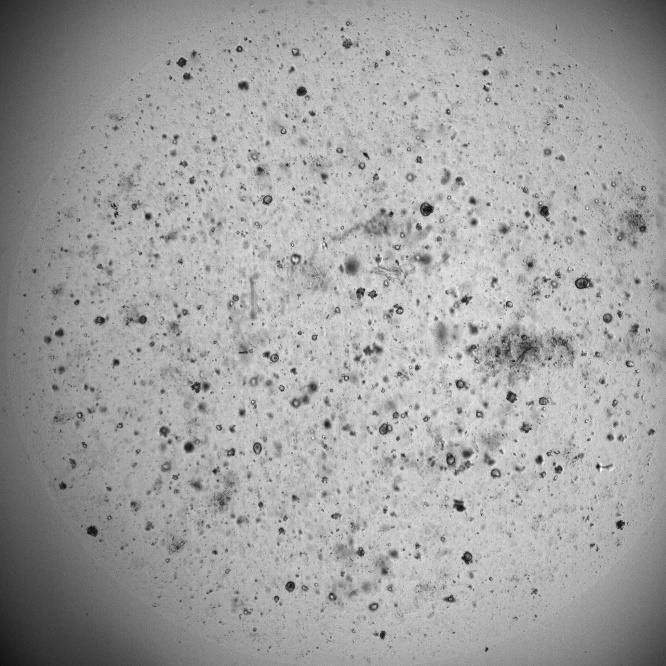

Supplement: Supplementary Figure S2 — Illustration of relative total area [file crc-25-0473_supplementary_figure_s2_suppsf2.docx]
