# Supplementary material for: A Classifier for Patient-Derived Colorectal Tumoroid Drug Sensitivity Using Confocal Imaging and Growth Rate Inhibition Metrics
Source: Cancer Res Commun. 2026 Mar 4;6(3):466–76. doi: 10.1158/2767-9764.CRC-25-0473 (PMC13012007; doi:10.1158/2767-9764.CRC-25-0473)
Supplement: Supplementary Figure S3 — Time-course analysis of sample library dose-response with raw measurements and standard deviation included. [file crc-25-0473_supplementary_figure_s3_suppfs3.docx]

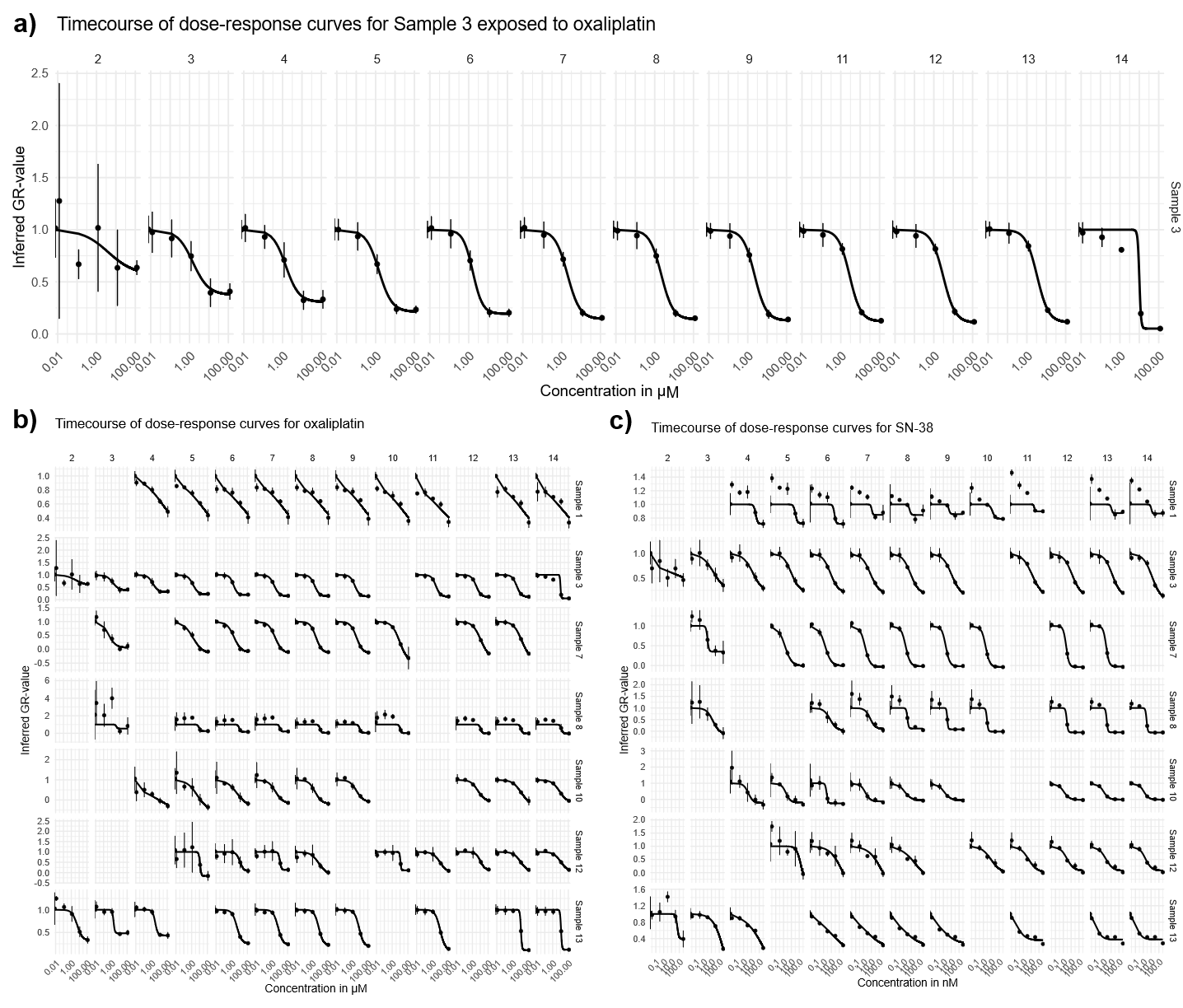


**Supplementary Figure S3.** Time-course analysis of sample library dose-response with raw measurements and standard deviation included. a) Time-course of dose-response curves for Sample 3 exposed to oxaliplatin, b-c) Time-course of dose-response curves for 7 samples cultivated for 13-14 days. Blank spaces indicate either missing data from that day, or that a curve could not be fit to data from that day.
