# Supplementary material for: A Classifier for Patient-Derived Colorectal Tumoroid Drug Sensitivity Using Confocal Imaging and Growth Rate Inhibition Metrics
Source: Cancer Res Commun. 2026 Mar 4;6(3):466–76. doi: 10.1158/2767-9764.CRC-25-0473 (PMC13012007; doi:10.1158/2767-9764.CRC-25-0473)
Supplement: Supplementary Figure S4 — Time course analysis of point estimate GR50 for 7 samples cultivated for a total of 13-14 days. [file crc-25-0473_supplementary_figure_s4_suppsf4.docx]

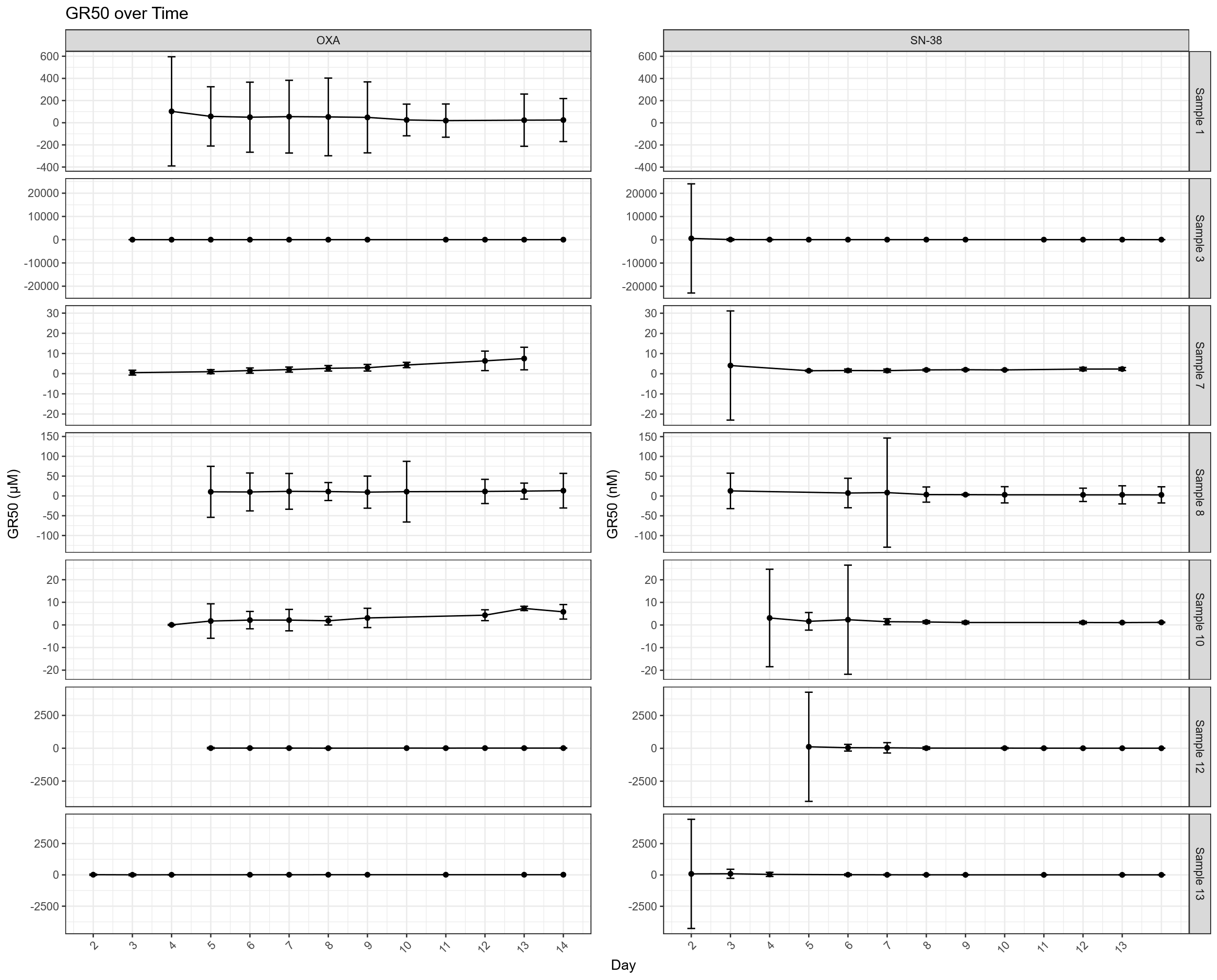


**Supplementary Figure S4.** Time course analysis of point estimate GR50 for 7 samples cultivated for a total of 13-14 days. Missing dots indicate either missing data from that day, or that a dose-response curve could not be fit to the specific day. Error-bars indicate the standard error of the estimate. Y axes are linear.
