# Supplementary material for: A Classifier for Patient-Derived Colorectal Tumoroid Drug Sensitivity Using Confocal Imaging and Growth Rate Inhibition Metrics
Source: Cancer Res Commun. 2026 Mar 4;6(3):466–76. doi: 10.1158/2767-9764.CRC-25-0473 (PMC13012007; doi:10.1158/2767-9764.CRC-25-0473)
Supplement: Supplementary Figure S5 — Classification of Samples 1, 3, 7, 8, 10, 12, and 13 as either sensitive or resistant to oxaliplatin and SN-38 for the total duration of the experiment, according to the median GR50 of all 16 samples on day 7 of the experiment. [file crc-25-0473_supplementary_figure_s5_suppsf5.docx]

|  | 2 | 3 | 4 | 5 | 6 | 7 | 8 | 9 | 10 | 11 | 12 | 13 | 14 |
| --- | --- | --- | --- | --- | --- | --- | --- | --- | --- | --- | --- | --- | --- |
| Sample 1 |  |  |  |  |  |  |  |  |  |  |  |  |  |
| Oxaliplatin |  |  |  |  |  |  |  |  |  |  |  |  |  |
| SN-38 |  |  |  |  |  |  |  |  |  |  |  |  |  |
| Sample 3 |  |  |  |  |  |  |  |  |  |  |  |  |  |
| Oxaliplatin |  |  |  |  |  |  |  |  |  |  |  |  |  |
| SN-38 |  |  |  |  |  |  |  |  |  |  |  |  |  |
| Sample 7 |  |  |  |  |  |  |  |  |  |  |  |  |  |
| Oxaliplatin |  |  |  |  |  |  |  |  |  |  |  |  |  |
| SN-38 |  |  |  |  |  |  |  |  |  |  |  |  |  |
| Sample 8 |  |  |  |  |  |  |  |  |  |  |  |  |  |
| Oxaliplatin |  |  |  |  |  |  |  |  |  |  |  |  |  |
| SN-38 |  |  |  |  |  |  |  |  |  |  |  |  |  |
| Sample 10 |  |  |  |  |  |  |  |  |  |  |  |  |  |
| Oxaliplatin |  |  |  |  |  |  |  |  |  |  |  |  |  |
| SN-38 |  |  |  |  |  |  |  |  |  |  |  |  |  |
| Sample 12 |  |  |  |  |  |  |  |  |  |  |  |  |  |
| Oxaliplatin |  |  |  |  |  |  |  |  |  |  |  |  |  |
| SN-38 |  |  |  |  |  |  |  |  |  |  |  |  |  |
| Sample 13 |  |  |  |  |  |  |  |  |  |  |  |  |  |
| Oxaliplatin |  |  |  |  |  |  |  |  |  |  |  |  |  |
| SN-38 |  |  |  |  |  |  |  |  |  |  |  |  |  |

**Supplementary Figure S5.** Classification of Samples 1, 3, 7, 8, 10, 12, and 13 as either sensitive or resistant to oxaliplatin and SN-38 for the total duration of the experiment, according to the median GR50 of all 16 samples on day 7 of the experiment. Red = resistant, green = sensitive, grey = missing data from that day of the experiment, or no curve could be fit to data from that day of the experiment.
