# Supplementary material for: A Classifier for Patient-Derived Colorectal Tumoroid Drug Sensitivity Using Confocal Imaging and Growth Rate Inhibition Metrics
Source: Cancer Res Commun. 2026 Mar 4;6(3):466–76. doi: 10.1158/2767-9764.CRC-25-0473 (PMC13012007; doi:10.1158/2767-9764.CRC-25-0473)
Supplement: Supplementary Figure S6 — Growth rate (relative total area) of negative controls in all growing samples. [file crc-25-0473_supplementary_figure_s6_suppsf6.docx]

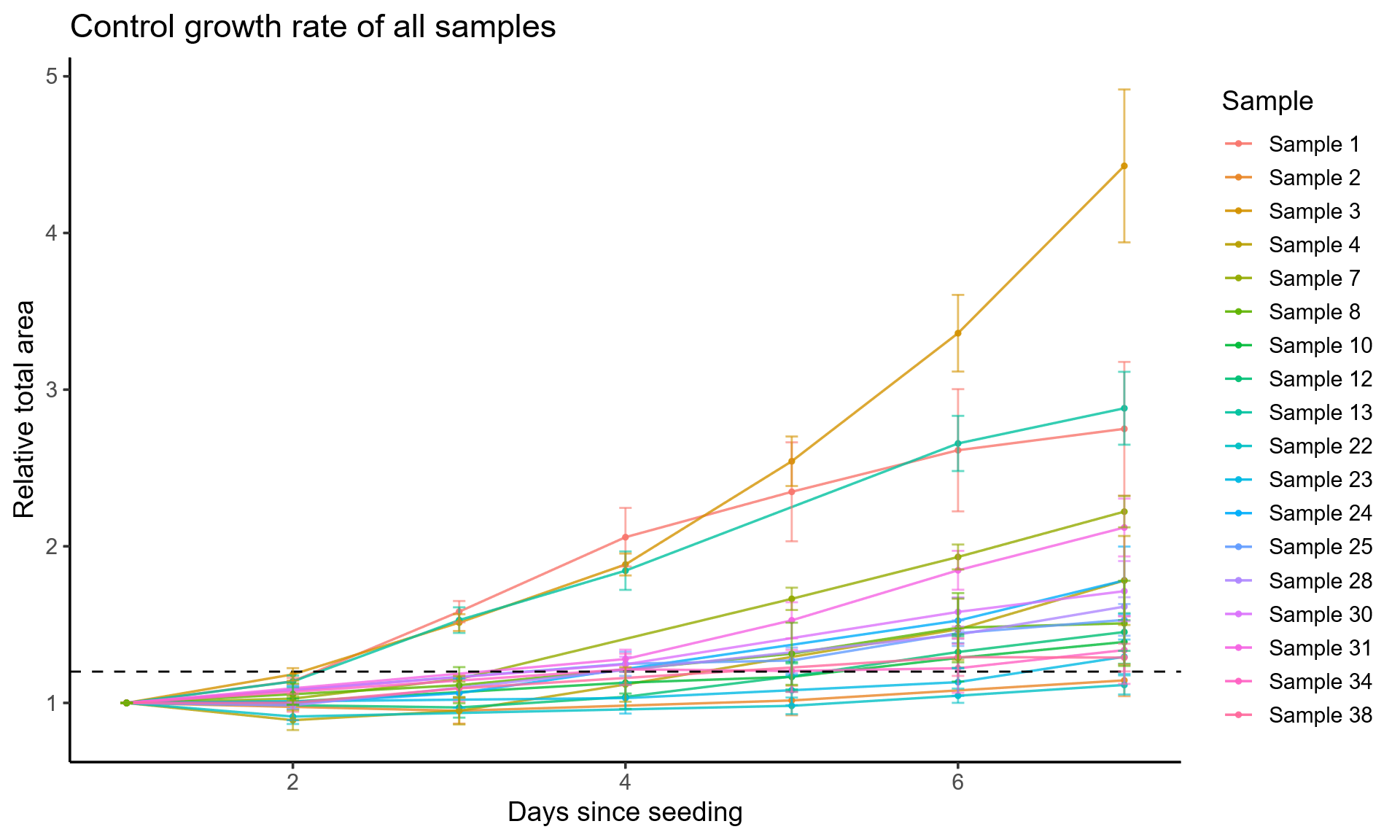


**Supplementary Figure S6.** Growth rate (relative total area) of negative controls in all growing samples. Dashed black line represents a growth rate of 1.2, the predetermined level of growth rate that must be achieved by day 7 in order to define a sample as successful. Errorbars = +/- SD.
