# Supplementary material for: A Classifier for Patient-Derived Colorectal Tumoroid Drug Sensitivity Using Confocal Imaging and Growth Rate Inhibition Metrics
Source: Cancer Res Commun. 2026 Mar 4;6(3):466–76. doi: 10.1158/2767-9764.CRC-25-0473 (PMC13012007; doi:10.1158/2767-9764.CRC-25-0473)
Supplement: Supplementary Figure S7 — Dose response curves for Oxaliplatin generated using the log-logistic 3-parametric model fitted to either relative total area or GR-metrics. [file crc-25-0473_supplementary_figure_s7_suppsf7.docx]

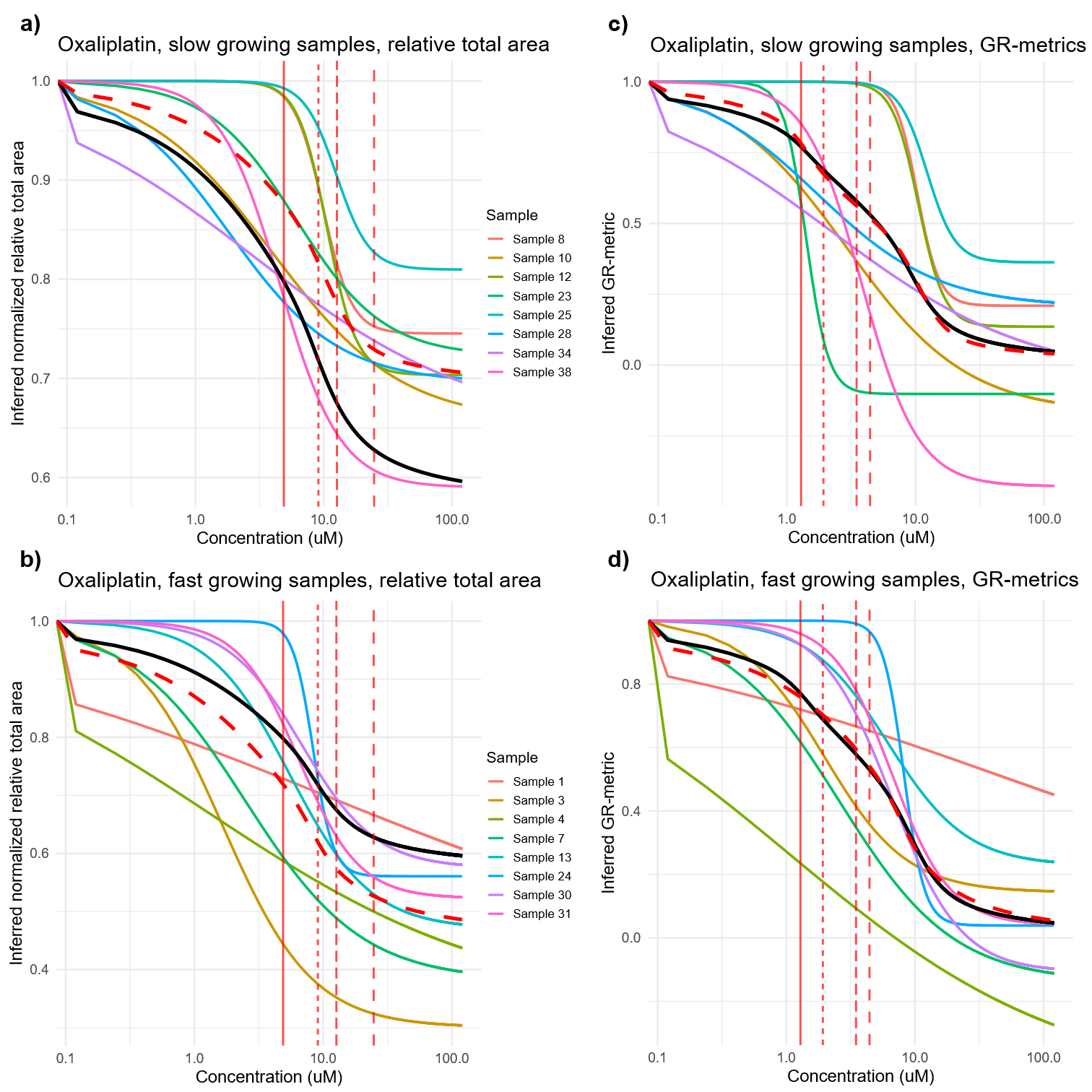


**Supplementary Figure S7. Dose response curves for Oxaliplatin generated using the log-logistic 3-parametric model fitted to either relative total area (a-b) or GR-metrics (c-d).** X-axis displays drug concentration in micromolar (µM) and y-axis displays inferred response (normalized relative total area/GR-metric). Solid black line indicates mean inferred response across both fast- and slow growing samples pr. readout, and dotted red line indicates the mean inferred response for only the fast or slow growing samples, respectively, pr. readout. Vertical red lines indicate median ED-values for increasing levels of inhibition (20%, 30%, 40%, and 50%) across both fast- and slow growing samples. **a)** Dose-response curves of the 8 slowest growing samples fitted to relative total area. **b)** Dose-response curves of the 8 fastest growing samples fitted to relative total area. **c)** Dose-response curves of the 8 slowest growing samples fitted to GR-metrics. **d)** Dose-response curves of the 8 fastest growing samples fitted to GR-metrics.
