# Supplementary material for: A Classifier for Patient-Derived Colorectal Tumoroid Drug Sensitivity Using Confocal Imaging and Growth Rate Inhibition Metrics
Source: Cancer Res Commun. 2026 Mar 4;6(3):466–76. doi: 10.1158/2767-9764.CRC-25-0473 (PMC13012007; doi:10.1158/2767-9764.CRC-25-0473)
Supplement: Supplementary Figure S8 — Classification of the eight slowest growing samples as either sensitive or resistant to oxaliplatin and SN-38 at day 7 of the experiment according to either median ED20 or median GR50. [file crc-25-0473_supplementary_figure_s8_suppsf8.docx]

|  | **Relative total area/median ED20** | **GR-metrics/median GR50** |
| --- | --- | --- |
| **Sample 8** |  |  |
| Oxaliplatin |  |  |
| SN-38 |  |  |
| **Sample 10** |  |  |
| Oxaliplatin |  |  |
| SN-38 |  |  |
| **Sample 12** |  |  |
| Oxaliplatin |  |  |
| SN-38 |  |  |
| **Sample 23** |  |  |
| Oxaliplatin |  |  |
| SN-38 |  |  |
| **Sample 25** |  |  |
| Oxaliplatin |  |  |
| SN-38 |  |  |
| **Sample 28** |  |  |
| Oxaliplatin |  |  |
| SN-38 |  |  |
| **Sample 34** |  |  |
| Oxaliplatin |  |  |
| SN-38 |  |  |
| **Sample 38** |  |  |
| Oxaliplatin |  |  |
| SN-38 |  |  |

**Supplementary figure S8.** Classification of the eight slowest growing samples as either sensitive or resistant to oxaliplatin and SN-38 at day 7 of the experiment according to either median ED20 (derived from dose-response curves fitted to relative total area) or median GR50 (derived from dose-response curves fitted to GR-metrics). Red = resistant, green = sensitive.
