# Supplementary material for: A Classifier for Patient-Derived Colorectal Tumoroid Drug Sensitivity Using Confocal Imaging and Growth Rate Inhibition Metrics
Source: Cancer Res Commun. 2026 Mar 4;6(3):466–76. doi: 10.1158/2767-9764.CRC-25-0473 (PMC13012007; doi:10.1158/2767-9764.CRC-25-0473)
Supplement: Supplementary Figure S10 — Barplot of estimated GR50 of simulated samples. [file crc-25-0473_supplementary_figure_s10_suppsf10.docx]

**
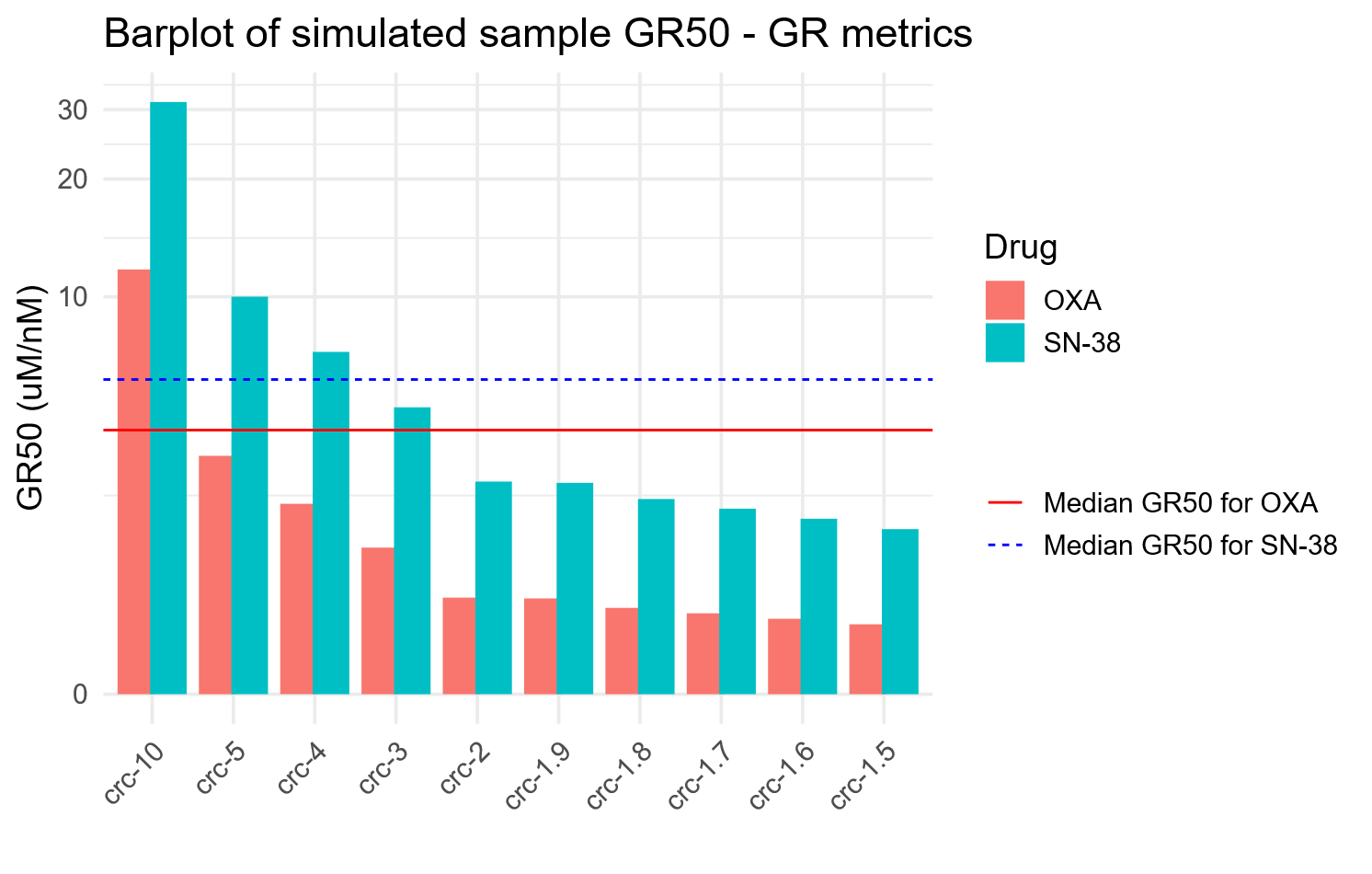
**

**Supplementary Figure S10.** Barplot of estimated GR50 of simulated samples. Sample name corresponds to growth rate, i.e. relative total area, on day 7 of the experiment. The solid red line represents median GR50 for oxaliplatin from our live samples, while the dashed blue line represents the median GR50 for SN-38 from our live samples. 9/10 simulated samples were classified as sensitive to oxaliplatin, and /10 samples were classified as sensitive to SN-38 using median GR50 as cutoff. Y axis is pseudo-log transformed. GR50 is µM for oxaliplatin and nM for SN-38.
