# Supplementary material for: A Classifier for Patient-Derived Colorectal Tumoroid Drug Sensitivity Using Confocal Imaging and Growth Rate Inhibition Metrics
Source: Cancer Res Commun. 2026 Mar 4;6(3):466–76. doi: 10.1158/2767-9764.CRC-25-0473 (PMC13012007; doi:10.1158/2767-9764.CRC-25-0473)
Supplement: Supplementary Figure S11 — Barplot of estimated ED20 of simulated samples. [file crc-25-0473_supplementary_figure_s11_suppsf11.docx]

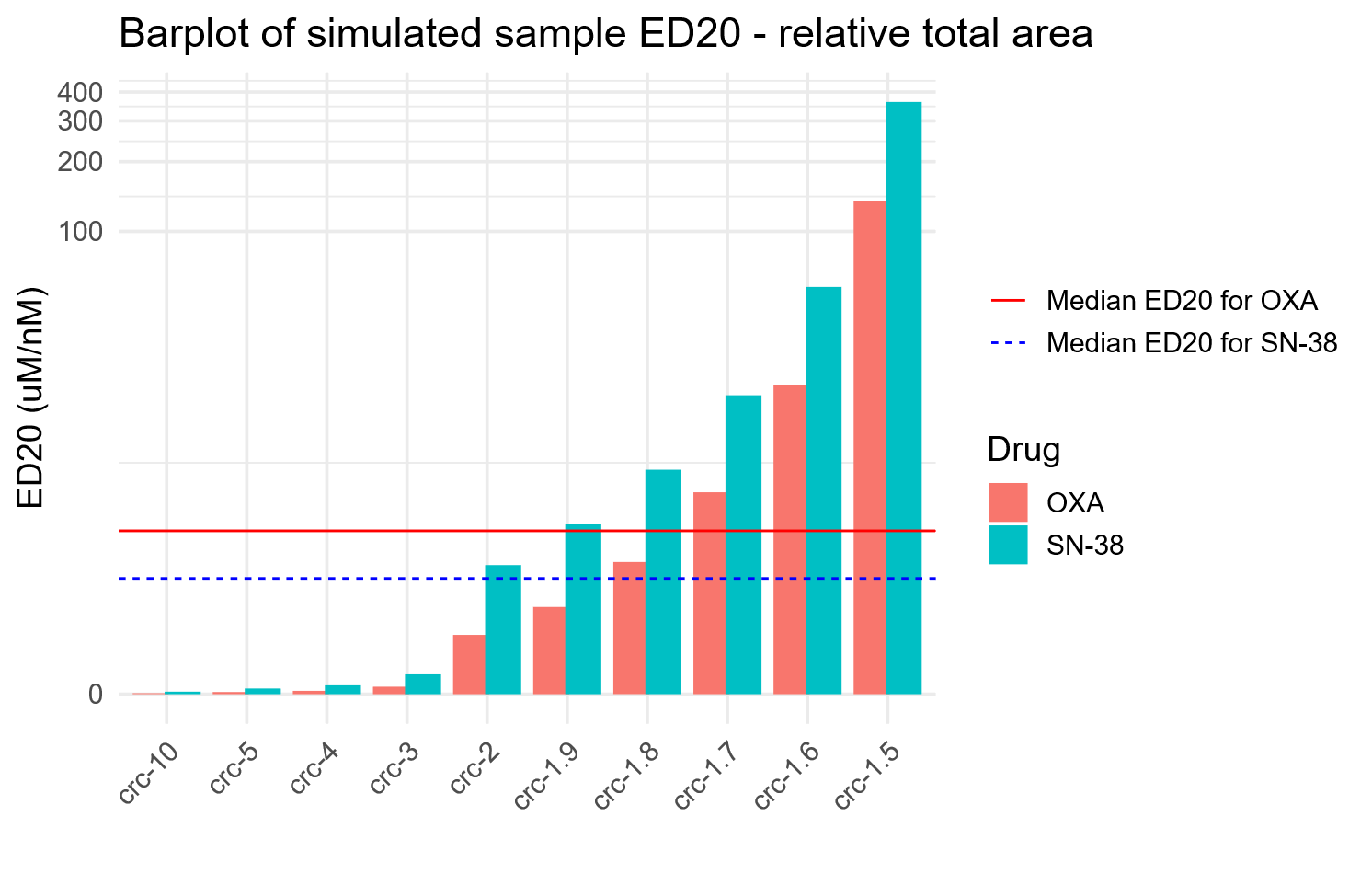


**Supplementary Figure S11.** Barplot of estimated ED20 of simulated samples. Sample name corresponds to growth rate, i.e. relative total area, on day 7 of the experiment. The solid red line represents median ED20 for oxaliplatin from our live samples, while the dashed blue line represents the median ED20 for SN-38 from our live samples. 7/10 simulated samples were classified as sensitive to oxaliplatin, and 4/10 samples were classified as sensitive to SN-38 using median GR50 as cutoff. Y-axis is pseudo-log transformed. GR50 is µM for oxaliplatin and nM for SN-38.
