# Supplementary material for: A Classifier for Patient-Derived Colorectal Tumoroid Drug Sensitivity Using Confocal Imaging and Growth Rate Inhibition Metrics
Source: Cancer Res Commun. 2026 Mar 4;6(3):466–76. doi: 10.1158/2767-9764.CRC-25-0473 (PMC13012007; doi:10.1158/2767-9764.CRC-25-0473)
Supplement: Supplementary Figure S12 — Sensitivity of all samples as sensitive or resistant to oxaliplatin and SN-38 using median GR50 and median AOC. [file crc-25-0473_supplementary_figure_s12_suppsf12.docx]

|  | **GR50 – estimate (SD)** | **AOC - estimate** |
| --- | --- | --- |
| **Sample 1** |  |  |
| Oxaliplatin | 54.7 (-273 – 383) µM | 58.5 |
| SN-38 | NA | 47.2 |
| **Sample 3** |  |  |
| Oxaliplatin | 2.54 (1.34 – 3.73) µM | 98.5 |
| SN-38 | 13.7 (-1.00 – 28.3) nM | 229 |
| **Sample 4** |  |  |
| Oxaliplatin | 0.20 (-0.07 – 0.47) µM | 142 |
| SN-38 | 2.20 (-20.0 – 24.0) nM | 316 |
| **Sample 7** |  |  |
| Oxaliplatin | 2.01 (0.74 – 3.29) µM | 125 |
| SN-38 | 1.50 (0.72 – 2.27) nM | 326 |
| **Sample 8** |  |  |
| Oxaliplatin | 11.5 (-33.7 – 56.7) µM | 86.1 |
| SN-38 | 8.33 (-129 – 146) nM | 272 |
| **Sample 10** |  |  |
| Oxaliplatin | 2.12 (-2.62– 6.86) µM | 126 |
| SN-38 | 1.41 (0.09 – 2.73) nM | 354 |
| **Sample 12** |  |  |
| Oxaliplatin | 11.3 (-3.51– 26.2) µM | 93.8 |
| SN-38 | 33.4 (-360 – 426) nM | 251 |
| **Sample 13** |  |  |
| Oxaliplatin | 9.50 (3.85 - 15.2) µM | 82.7 |
| SN-38 | 6.08 (-34.8 – 47.0) nM | 219 |
| **Sample 23** |  |  |
| Oxaliplatin | 1.36 (-23.8 – 26.5) µM | 131 |
| SN-38 | 2.26 (-1.29 – 5.81) nM | 344 |
| **Sample 24** |  |  |
| Oxaliplatin | 8.32 (-53.0 – 69.7) µM | 107 |
| SN-38 | 3.09(-3.51 – 9.69) nM | 268 |
| **Sample 25** |  |  |
| Oxaliplatin | 17.1 (-94.5– 129) µM | 67.8 |
| SN-38 | 65.2 (-276 – 406) nM | 208 |
| **Sample 28** |  |  |
| Oxaliplatin | 3.07 (-2.63 – 8.77) µM | 88.7 |
| SN-38 | 115 (-1086 – 1316) nM | 168 |
| **Sample 30** |  |  |
| Oxaliplatin | 5.80 (-9.61– 21.2) µM | 120 |
| SN-38 | 2.31 (-31.2 – 35.8) nM | 256 |
| **Sample 31** |  |  |
| Oxaliplatin | 6.88 (3.01 – 10.7) µM | 105 |
| SN-38 | 7.18 (2.82 – 11.5) nM | 264 |
| **Sample 34** |  |  |
| Oxaliplatin | 1.84 (-7.36– 11.0) µM | 105 |
| SN-38 | 9.92 (-104– 124) nM | 212 |
| **Sample 38** |  |  |
| Oxaliplatin | 2.80 (-4.30 – 9.89) µM | 163 |
| SN-38 | 0.37 (-1.46 – 2.20) nM | 365 |

**Supplementary Figure S12.** Sensitivity of all samples as sensitive or resistant to oxaliplatin and SN-38 using median GR50 and median AOC. Red = resistant, green = sensitive, NA = GR50 could not be estimated.
