# Supplementary material for: A Classifier for Patient-Derived Colorectal Tumoroid Drug Sensitivity Using Confocal Imaging and Growth Rate Inhibition Metrics
Source: Cancer Res Commun. 2026 Mar 4;6(3):466–76. doi: 10.1158/2767-9764.CRC-25-0473 (PMC13012007; doi:10.1158/2767-9764.CRC-25-0473)
Supplement: Supplementary Material — Detailed description of methods. [file crc-25-0473_supplementary_material_suppsm.docx]

# Supplementary material

Development of a classifier for drug sensitivity in patient-derived colorectal tumoroids using confocal imaging and growth-rate metrics

Baard Cristoffer Sakshaug^1^, Tonje Husby Haukaas^2^, Evelina Folkesson^1,2^, Christa Ringers^1^, Henri Colyn Harry Bwanika^1^, Margrét Sylvía Sigfúsdóttir^2^, Hanne Hein Trøen^2^, Sigri Bakken Sperstad^2^, Ingrid Aune Bergstrøm^1^, Tore Stornes^4^, Geir Klinkenberg^2^, Torkild Visnes^2^, Åsmund Flobak^1,2,3^

1. Department of Clinical and Molecular Medicine, Norwegian University of Science and Technology, Trondheim, Norway
2. Department of Biotechnology and Nanomedicine, SINTEF Industry, Trondheim, Norway
3. The Cancer Clinic, St Olav’s University Hospital, Trondheim, Norway
4. Department of Surgery, St. Olav’s University Hospital, Trondheim, Norway

Keywords: colorectal cancer, patient-derived tumouroids, chemotherapy, imaging, personalised medicine

# Details regarding Materials and methods

## Patient material

Human tumor specimens were collected immediately following surgical resection or biopsy from colorectal cancer patients, in accordance with institutional ethical approvals and informed consent. Upon retrieval, tissue samples were placed in 20 mL of supplemented IntestiCult^TM^ Organoid Growth Medium (Human, Cat. # 06010, STEMCELL Technologies) or DMEM (Cat. # D5671, Sigma Aldrich containing 15 µL ROCK inhibitor Y-27632 (Cat. # SCM075 Merck Millipore), to a final concentration of 10 µM final in 50-mL centrifuge tube. Samples were kept on ice or at 4 °C until further processing, which was initiated as soon as possible to preserve tissue viability.

## Sample processing

## A 50-mL centrifuge tube containing 20 mL of supplemented IntestiCult^TM^ or DMDEM and 15 µL ROCK inhibitor was pre-weighed before adding the tissue sample. After the sample was added, the tube was weighed again to determine the weight of the tissue. The storage medium was discarded, and the sample was washed with 20 mL of HBSS by gently inverting the tube. The wash was removed, and 20 mL of fresh HBSS was added. The tissue and medium were transferred to a 10-cm tissue culture dish. Necrotic tissue was removed using forceps or a sterile razor blade. The viable tumor material was divided into smaller portions: one piece was snap-frozen in liquid nitrogen, another was fixed in formalin, a third was cryopreserved in 10% DMSO-FBS, and the remaining tissue was processed for the establishment of tumoroid cultures. The piece designated for culture was transferred to a fresh 10-cm dish containing 30 mL HBSS and minced finely using sterile forceps or razor blades until fragments were 1–2 mm in size. The minced tissue was collected into a 50-mL tube, and the culture dish was rinsed with 20 mL HBSS to recover any residual tissue. The suspension was centrifugated at 400–600 × g at 4 °C for 5 minutes, and the supernatant was discarded. A second wash with 20 mL HBSS was performed. The pellet was resuspended in 19 mL IntestiCult^TM^ medium supplemented with 1 mL collagenase type II (Cat. # 17101015, Gibco) to a final concentration of 1 mg/mL and 15 µL ROCK inhibitor. Mechanical digestion was carried out by passing the sample through pre-wetted syringes equipped with 1.4 mm and 1.2 mm needles. The suspension was then transferred to a 100-mL sterile conical flask containing a magnetic stir bar and placed in a 37 °C water bath with constant stirring. Every 10 minutes, the flask was removed and the suspension pipetted vigorously using a 1-mL filter tip. This cycle was repeated until the tissue was sufficiently dissociated to pass through a 1-mL pipette tip. The digestion mixture was transferred to a 50-mL centrifuge tube and centrifugated at 400–600 × g at 4 °C for 5 minutes. The supernatant was discarded, and the pellet was resuspended in 20 mL HBSS.

## Tissue was considered sufficiently processed when it passed through a 0.6 mm needle. The suspension was filtered with a 500 µm PluriStrainer to remove large tissue fragments and debris, followed by filtration through a 300 µm PluriStrainer to collect intermediate-sized organoid fragments. The 500-300 µm retentate was washed into a fresh tube and optionally subjected to further digestion for higher yield. The filtrate (≤ 300 µm) was subsequently passed through a 40 µm PluriStrainer to isolate the tumoroid population of interest (300-40 µm). The strainer was dipped into a 10-cm dish containing 30 mL HBSS and gently swirled to release cells and small debris. It was then transferred to a fresh HBSS dish, and the retained organoids were collected using a 1-mL pipette. Samples were categorized by size: >300 µm (large tissue fragments for storage), 40–300 µm (tumoroid-rich fraction), and <40 µm (single-cell mix). Each fraction was centrifugated at 600 × g at 4 °C for 5 minutes. If pelleting was inadequate, a second centrifugation step was performed, with speed increased to 800 × g if necessary. The resulting tumoroid-rich fraction pellet was resuspended in fresh IntestiCult^TM^ or StemPro^TM^ human embryonic stem cell serum-free medium (SFSCM), mixed thoroughly and counted by loading 10 µL onto a C-chip counting slide. For Matrigel embedding, the tumoroid suspension was diluted to the desired concentration and mixed 1:1 with cold Matrigel (Cat. # 356231, Corning) using frozen wide-bore pipette tips and seeded in 50 µL droplets at a density of approximately 500 tumoroid/droplet in a flat bottom 24-well plate (Cat. # 3524, Corning). Matrigel tubes were kept on ice throughout. Plates were incubated at 37 °C for 30 minutes to solidify the Matrigel. Following polymerization, 500 µL of pre-warmed IntestiCult^TM^ or SFSCM was gently added along the wall of each well using a pipette tip, taking care not to disturb the gel.

## Drug exposure protocol

Following the day after seeding, growth medium was replaced with fresh medium containing Oxaliplatin (Cat. # S1224, Selleckchem), SN-38 (Cat. # S4908, Selleckchem) or the combination of the two drugs in 4 technical replicates. All stock concentration of drugs were dissolved in 100% DMSO and wells across the plate were DMSO normalized to a final concentration of 0.5%. Drugs were serially diluted in a 5-step, 10-fold concentration gradient ranging from 0.032 - 320 nM for SN-38 and 0.012 - 120 µM for Oxaliplatin. Imaging, as described below, was performed daily for a minimum of 7 days, and growth was calculated. For samples 1, 3, 7, 8, 10, 12, and 13, following 6-7 days of exposure, the medium was replaced with fresh IntestiCult^TM^ or SFSCM, and cultivation continued for an additional 6-7 days.

## Image acquisition and analysis

Using an ImageXpress Micro Confocal High-Content System, the growth of the spheroids was monitored daily for the duration of the experiment. The microscope was heated to 37 °C, and a Z-stack of images of each well were captured automatically. For each plate, a Z-stack interval was defined so that all spheroids in the gel droplets were imaged. After imaging, each stack of images was converted into a 2D maximum intensity projection image using a “best-focus” protocol. 2D-projection images were exported as TIFF files, which were further analyzed using the ImageJ software and a macro script (see below) performing automated format conversion, background subtraction, binarization and particle analysis. Before analysis, images were visually inspected to confirm growth and subsequently subjected to a testing script which varied the “rolling” variable in the run(“Subtract Background…”) argument, as well as the “saturated” variable in the run(“Enhance Contrast…”) argument. The former took on the values 10, 50, and 100, while the latter took on the values 0.5, 1.5, 2.5, and 3.5. The values visually deemed to best fit the sample was then selected for processing of all the images. For some samples, the run(“Remove Outliers…”) argument had to be adjusted to remove larger objects due to a lot of debris in the images. Images from wells with bubbles in them were excluded only on the day they appeared, unless the bubble appeared on day 1, in which case the entire well was excluded from analysis, or the bubble was manually coloured black to be excluded from analysis if this was feasible without compromising evaluation of tumoroids. Wells with broken gels were excluded from analysis from the first day they were observed to be broken in images. Wells with signs of infection, or significant amounts of non-tumouroid debris, were excluded from analysis from the day the signs of infection or debris appeared. For each image (well) the total area covered by spheroids (and other parameters) was calculated automatically. These data were exported and further processed using R version 4.4.2.

#
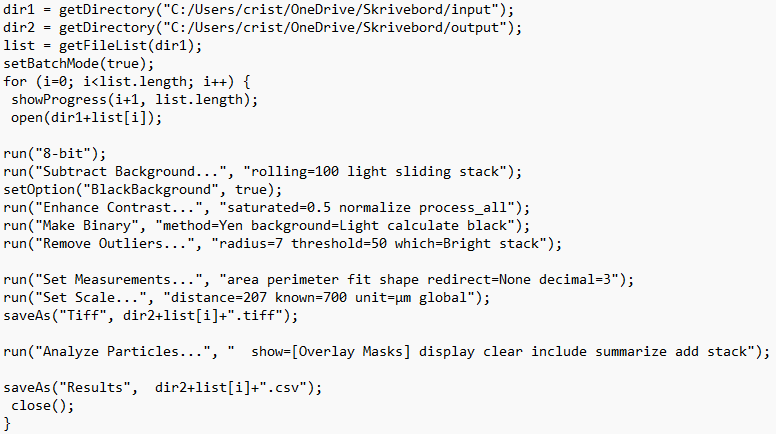


## Data analysis

See separately attached R-notebook file.
