# Supplementary material for: A Classifier for Patient-Derived Colorectal Tumoroid Drug Sensitivity Using Confocal Imaging and Growth Rate Inhibition Metrics
Source: Cancer Res Commun. 2026 Mar 4;6(3):466–76. doi: 10.1158/2767-9764.CRC-25-0473 (PMC13012007; doi:10.1158/2767-9764.CRC-25-0473)
Supplement: Supplementary Table S2 — List over materials and reagents used. [file crc-25-0473_supplementary_table_s2_suppst2.docx]

**Supplementary Table S2.** List over materials and reagents used for preparation, cultivation, and treatment of patient-derived tumouroids.

| **Component** | **Reference number** | **Manufacturer** |
| --- | --- | --- |
| Amphotericin B | A2942 | Sigma Aldrich |
| bFGF | 13256-029 | Invitrogen |
| Collagenase type II | 17101015 | Gibco |
| Dulbecco's Modified Eagle Medium (DMEM) | D5671 | Sigma Aldrich |
| Fetal Bovine Serum (FBS) | F7524 | Sigma Aldrich |
| 500 μm filters | 43-50500-50 | pluriSelect |
| 300 μm filters | 43-50300-03 | pluriSelect |
| 40 μm filters | 431750 | Corning |
| Hanks' Balanced Salt Solution (HBSS) | H9269 | Sigma Aldrich |
| Matrigel | 356231 | Corning |
| 2-mercaptoethanol | 21985023 | Gibco |
| Penicillin-Streptomycin | 15140-122 | Gibco |
| SN-38 | S4908 | Selleckchem |
| StemPro™ hESC SFM | A10006-01 | Gibco |
| 24-well flat bottom cultivation plates | 3524 | Corning |
| Y-27632 (ROCK inhibitor) | SCM075 | Sigma Aldrich |
| adDMEMF12 | 12634-010 | ThermoFischer |
| B27 | 12634-010 | ThermoFischer |
| GlutaMAX | 35050-061 | ThermoFischer |
| IntestiCult^TM^ Organoid Growth Medium (Human) | 06010 | Stemcell Technologies |
| Oxaliplatin | S1224 | Selleckchem |
