# Supplementary material for: A Classifier for Patient-Derived Colorectal Tumoroid Drug Sensitivity Using Confocal Imaging and Growth Rate Inhibition Metrics
Source: Cancer Res Commun. 2026 Mar 4;6(3):466–76. doi: 10.1158/2767-9764.CRC-25-0473 (PMC13012007; doi:10.1158/2767-9764.CRC-25-0473)
Supplement: Supplementary Table S3 — Reagents used for preparation of SFSCM. [file crc-25-0473_supplementary_table_s3_suppst3.docx]

**Supplementary Table S3** Reagents used for preparation of SFSCM

|  | **Concentration** | **Amount** |
| --- | --- | --- |
| DMEM/F12 + Glutamax-I | 1 X | 15.8925 mL |
| StemPro hESC Supplement | 1 X | 0.4 mL |
| BSA 25% | 1.8 % | 1.44 mL |
| FGFb (10μ/mL) | 8 ng/mL | 16 μL |
| 2-Mercaptoethanol (55mM) | 0.1 mM | 36.4 μL |
| Penicillin-streptomycin (10 000U/mL) | 100 U/mL | 0.2mL |
| Amphothericin B (250 μg/mL) | 25 μg/mL | 2 mL |
| ROCK-inhibitor (12.9 mM) | 10 μM | 15.5 μL |
