# Supplementary material for: A Classifier for Patient-Derived Colorectal Tumoroid Drug Sensitivity Using Confocal Imaging and Growth Rate Inhibition Metrics
Source: Cancer Res Commun. 2026 Mar 4;6(3):466–76. doi: 10.1158/2767-9764.CRC-25-0473 (PMC13012007; doi:10.1158/2767-9764.CRC-25-0473)
Supplement: Supplementary Table S4 — Reagents used for preparation of IntestiCult. [file crc-25-0473_supplementary_table_s4_suppst4.docx]

**Supplementary Table S4** Reagents used for preparation of IntestiCult^TM^.

|  | **Concentration** | **Amount** |
| --- | --- | --- |
| IntestiCult™ OGM Human Basal Medium | 1X | 8.895 mL |
| Organoid Supplement | 1X | 8.895 mL |
| Penicillin-streptomycin (10 000U/mL) | 100 U/mL | 200 μL |
| ROCK-inhibitor (12.9 mM) | 10 μM | 15 μL |
| Amphothericin B (250 μg/mL) | 25 μg/mL | 2 mL |
