# Supplementary material for: A Classifier for Patient-Derived Colorectal Tumoroid Drug Sensitivity Using Confocal Imaging and Growth Rate Inhibition Metrics
Source: Cancer Res Commun. 2026 Mar 4;6(3):466–76. doi: 10.1158/2767-9764.CRC-25-0473 (PMC13012007; doi:10.1158/2767-9764.CRC-25-0473)
Supplement: Supplementary Table S5 — List of R-packages used. [file crc-25-0473_supplementary_table_s5_suppst5.docx]

| **Supplementary Table S5.** List of R-packages used. | |
| --- | --- |
| **Package** | **Version** |
| tidyverse | 2.0.0 |
| ggpubr | 0.6.0 |
| gridExtra | 2.3 |
| ggplot2 | 3.5.1 |
| drc | 3.0.1 |
| openxlsx | 4.1.7.1 |
| purr | 1.0.2 |
| dplyr | 1.1.4 |
| tidyr | 1.3.1 |
| plotly | 4.10.4 |
| patchwork | 1.3.0 |
| Exact2x2 | 1.6.9 |
| scales | 1.3.0 |
| boot | 1.3-32 |
