# Supplementary material for: A Classifier for Patient-Derived Colorectal Tumoroid Drug Sensitivity Using Confocal Imaging and Growth Rate Inhibition Metrics
Source: Cancer Res Commun. 2026 Mar 4;6(3):466–76. doi: 10.1158/2767-9764.CRC-25-0473 (PMC13012007; doi:10.1158/2767-9764.CRC-25-0473)
Supplement: Supplementary Table S6 — Descriptive statistics of patient material. [file crc-25-0473_supplementary_table_s6_suppst6.docx]

| Supplementary Table S6. Descriptive statistics of patient material. * = p-value ≤ 0.05. | | |
| --- | --- | --- |
|  | Sample cultivation | |
|  | **Successful (n = 16)** | **Unsuccessful (n = 19)** |
| Gender (m/f) | 13/3 | 10/9 |
| Age (mean/median) | 71/73 | 75/77 |
| Preoperative WHO-PS (0/1/2/3/4/5) | 15/1/0/0/0/0 | 16/2/1/0/0/0 |
| Tumor size (mm)  Mean (SD)  Median (IQR) | 49 (18.5)  49 (37.5 – 53) | 51 (23.0)  45 mm (39.5 – 60.5) |
| Tumor location (right/left/rectal)* | 6/10/0 | 13/4/2 |
| Preoperative CEA (µg/L)  Mean (SD)  Median (IQR | 5.8 (6.7)  2.9 (1.95 – 6.75) | 79.4 (212)  4.2 (1.6 - 20.1) |
| Histological grade (I/II/III) | 1/13/2 | 1/12/6 |
| Clinical stage (I/II/III/IV)* | 3/6/7/0 | 4/10/4/1 |
| Neoadjuvant treatment (y/n) | 0/16 | 0/19 |
| Adjuvant treatment (y/n)* | 6/10 | 1/18 |
| Recurrence (y/n) | 1/15 | 1/18 |
| Palliative treatment (y/n) | 0/16 | 1/18 |
