# Supplementary material for: A Classifier for Patient-Derived Colorectal Tumoroid Drug Sensitivity Using Confocal Imaging and Growth Rate Inhibition Metrics
Source: Cancer Res Commun. 2026 Mar 4;6(3):466–76. doi: 10.1158/2767-9764.CRC-25-0473 (PMC13012007; doi:10.1158/2767-9764.CRC-25-0473)
Supplement: Supplementary Table S7 — Residual standard error (RSE) of all sample-drug combinations. [file crc-25-0473_supplementary_table_s7_suppst7.docx]

| Supplementary Table S7. Residual standard error (RSE) of all sample-drug combinations. | | |
| --- | --- | --- |
|  | **RSE** | |
|  | **Oxaliplatin** | **SN-38** |
| Sample 1 | 0.054 | 0.19 |
| Sample 3 | 0.023 | 0.028 |
| Sample 4 | 0.047 | 0.11 |
| Sample 7 | 0.046 | 0.043 |
| Sample 8 | 0.69 | 0.43 |
| Sample 10 | 0.14 | 0.085 |
| Sample 12 | 0.12 | 0.18 |
| Sample 13 | 0.033 | 0.065 |
| Sample 23 | 1.10 | 0.15 |
| Sample 24 | 0.10 | 0.36 |
| Sample 25 | 0.19 | 0.12 |
| Sample 28 | 0.073 | 0.12 |
| Sample 30 | 0.22 | 0.17 |
| Sample 31 | 0.039 | 0.025 |
| Sample 34 | 0.17 | 0.31 |
| Sample 38 | 0.22 | 0.14 |
